# PTPRJ Inhibits Leptin Signaling, and Induction of PTPRJ in the Hypothalamus Is a Cause of the Development of Leptin Resistance

**DOI:** 10.1038/s41598-017-12070-7

**Published:** 2017-09-14

**Authors:** Takafumi Shintani, Satoru Higashi, Ryoko Suzuki, Yasushi Takeuchi, Reina Ikaga, Tomomi Yamazaki, Kenta Kobayashi, Masaharu Noda

**Affiliations:** 1 Division of Molecular Neurobiology, National Institute for Basic Biology, Okazaki, 444-8787 Japan; 2 School of Life Science, The Graduate University for Advanced Studies (SOKENDAI), Okazaki, 444-8787 Japan; 3 Section of Major Nutrients, Department of Nutritional Science, National Institute of Health and Nutrition, National Institutes of Biomedical Innovation, Tokyo, 162-8636 Japan; 4 Section of Viral Vector Development, National Institute for Physiological Sciences, Okazaki, 444-8585 Japan

## Abstract

Leptin signaling in the hypothalamus plays a crucial role in the regulation of body weight. Leptin resistance, in which leptin signaling is disrupted, is a major obstacle to the improvement of obesity. We herein demonstrated that *protein tyrosine phosphatase receptor type J* (*Ptprj*) is expressed in hypothalamic neurons together with leptin receptors, and that PTPRJ negatively regulates leptin signaling by inhibiting the activation of JAK2, the primary tyrosine kinase in leptin signaling, through the dephosphorylation of Y813 and Y868 in JAK2 autophosphorylation sites. Leptin signaling is enhanced in *Ptprj*-deficient mice, and they exhibit lower weight gain than wild-type mice because of a reduced food intake. Diet-induced obesity and the leptin treatment up-regulated PTPRJ expression in the hypothalamus, while the overexpression of PTPRJ induced leptin resistance. Thus, the induction of PTPRJ is a factor contributing to the development of leptin resistance, and the inhibition of PTPRJ may be a potential strategy for improving obesity.

## Introduction

Obesity rates have been increasing at an explosive rate worldwide^1,2^. Obesity is now a global health issue because it is a major risk factor for cardiovascular diseases, type 2 diabetes, and some cancers^3,4^. The balance between food intake and energy expenditure has a direct influence on body weight, and the disruption of this energy homeostasis causes obesity. The hypothalamus in the brain senses nutritional states in the body through hormone and nutrient levels in blood and via the autonomic nervous system, and, consequently, modulates food intake and energy expenditure^5^. Leptin, an adipocyte-derived hormone, is considered to be the most critical factor controlling energy homeostasis; it regulates energy homeostasis through the inactivation of orexigenic neuropeptide Y (NPY)/agouti-related neuropeptide (AGRP) neurons and activation of anorexigenic proopiomelanocortin (POMC) neurons in the hypothalamic arcuate nucleus (ARC)^6^. Plasma leptin concentrations strongly correlate with body fat mass, and, hence, may be used as a biomarker of obesity^7,8^.

Leptin receptors are strongly expressed in ARC neurons in the hypothalamus^6^. The binding of leptins to long-form leptin receptors (LepRb) induces the dimerization of receptor molecules, and leads to the activation of the associated non-receptor-type tyrosine kinase, Janus kinase 2 (JAK2), by autophosphorylation^6,9,10^. Upon activation, JAK2 phosphorylates specific tyrosine residues in the intracellular tail of LepRb, which leads to the serial phosphorylation and activation of downstream signaling proteins, such as the signal transducer and activator of transcription 3 (STAT3)^11^. Phosphorylated STAT3 (pSTAT3) translocates to the nucleus, at which it regulates the transcriptional activity of various genes, including NPY, AGRP, POMC, and the signaling inhibitor suppressor of cytokine signaling 3 (SOCS3), by binding to their promoter regions^12^. SOCS3 constitutes an inhibitory feedback loop in leptin signaling by targeting the LepRb-JAK2 complex^13,14^.

Most obese individuals have an increased food intake despite high circulating leptin levels, which is referred to as leptin resistance^15^. Two mechanisms, a decrease in leptin transport from the periphery into the brain and the suppression of leptin signaling by an increase in negative regulatory molecules, have been proposed to explain central leptin resistance^16^. The cerebrospinal fluid (CSF)/serum leptin ratio is decreased in obesity^17^, and leptin transport by median eminence tanycytes is impaired in obese mice^18^. On the other hand, negative regulators for leptin signaling such as SOCS3 and protein tyrosine phosphatase 1B (PTP1B) were previously shown to be up-regulated in diet-induced obesity^19,20^. Other mechanisms, including hypothalamic inflammation and endoplasmic reticulum (ER) stress, are known to be involved in obesity-associated leptin resistance^16^. However, the exact mechanisms underlying leptin resistance in obese patients have yet to be elucidated.

Protein tyrosine phosphatases (PTPs) regulate signal transduction pathways involving tyrosine phosphorylation. The human genome encodes 107 PTPs, 38 of which are classical tyrosine-specific PTPs: among them, 20 members are transmembrane receptor-like PTPs (RPTPs) and 18 are intracellular PTPs^21^. RPTPs consist of an extracellular region, single transmembrane segment, and cytoplasmic region with one or two tyrosine phosphatase domains. RPTPs have been classified into eight subfamilies (R1/R6, R2a, R2b, R3, R4, R5, R7, and R8) based on the sequence homology of their extracellular and PTP domains^22^. Previous studies, including ours, demonstrated that some RPTPs negatively regulate the activation of receptor protein tyrosine kinases (RPTKs) through the dephosphorylation of their specific phosphorylated tyrosine residues^23–25^.

We recently reported that members of the R3 RPTP subfamily (PTPRB, PTPRH, PTPRJ, and PTPRO) dephosphorylate the insulin receptor (IR) as a substrate^26^. The R3 subfamily is characterized by fibronectin type III-like repeats in the extracellular region and a single PTP domain in the intracellular region. R3 RPTPs preferentially dephosphorylate specific phosphorylated tyrosine residues, Y960 and Y1146 (in mice), in the IR. Among R3 subfamily members, PTPRJ is expressed in insulin target tissues, such as the liver, adipose tissue, skeletal muscle, and brain^26^. *Ptprj*-deficient (*Ptprj*-KO) mice showed enhanced insulin signaling and improved glucose and insulin tolerance^26^. Thus, PTPRJ is an enzyme that attenuates insulin signaling *in vivo* ^26,27^.

We subsequently found that *Ptprj*-KO mice exhibited lower weight gain, associated with a lower food intake, than wild-type (WT) mice. In the present study, we investigated whether PTPRJ regulates leptin signaling in the hypothalamus. We revealed that PTPRJ negatively regulates leptin signaling by dephosphorylating specific tyrosine residues (Y813 and Y868) in JAK2, the simultaneous phosphorylation of which plays a pivotal role in JAK2 activation. *Ptprj*-KO mice exhibited enhanced leptin signaling: The stronger activation of STAT3 by phosphorylation and enhanced suppression of food intake by leptin administration in the brain. PTPRJ expression in the hypothalamus was up-regulated by diet-induced obesity or a leptin treatment, and, thus, diet-induced leptin resistance did not occur in *Ptprj*-KO mice. Furthermore, the overexpression of PTPRJ in the hypothalamus induced leptin resistance in lean mice.

## Results

### *Ptprj-*KO mice show a low food intake, and, thus, are markedly resistance to diet-induced obesity

**Figure 1:**
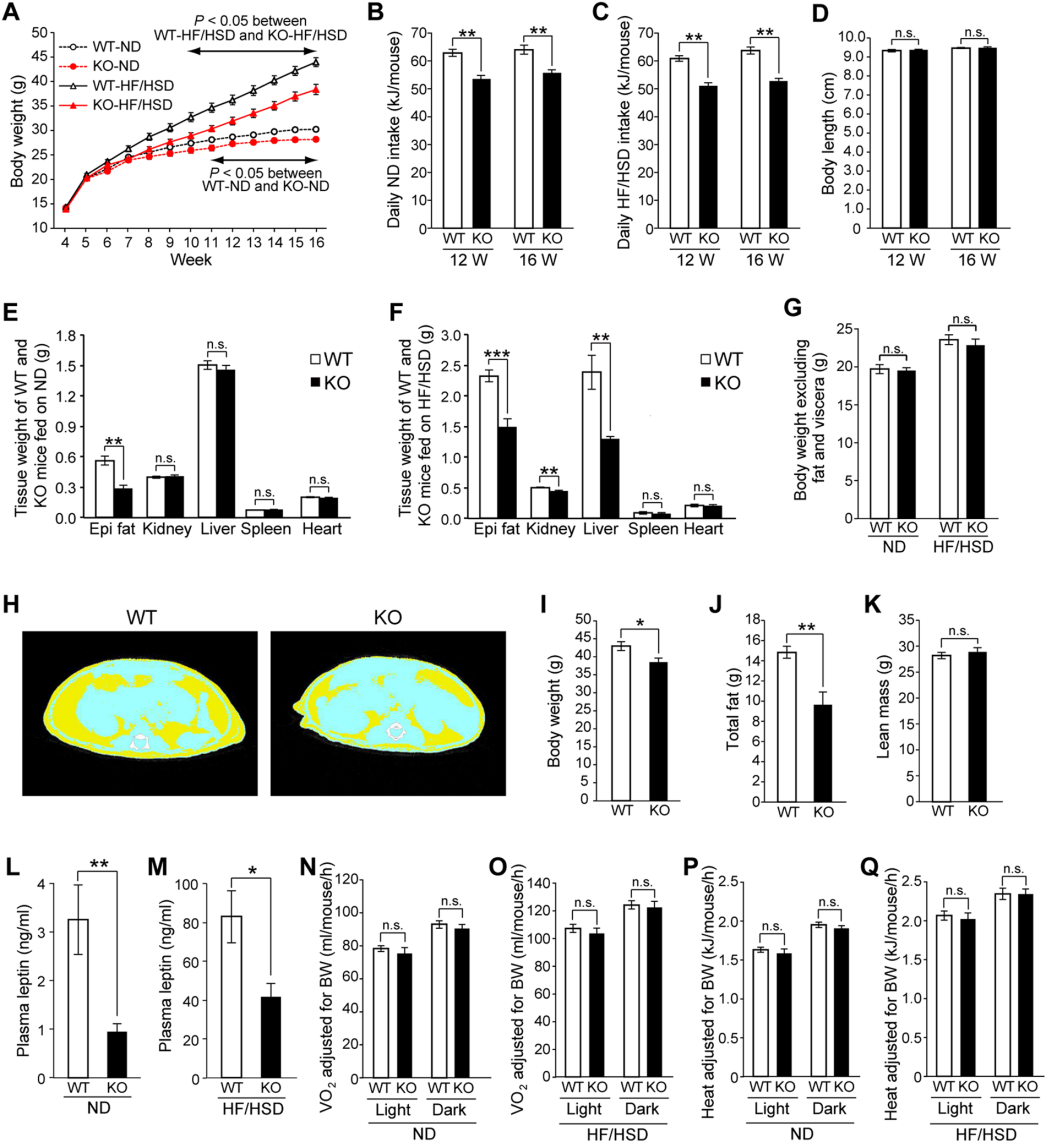
*Ptprj*-KO mice exhibit a lean phenotype and show resistance to diet-induced obesity. (**A**) Weekly body weights by wild-type (WT) and *Ptprj*-KO (KO) male mice fed ND (n = 11 each) or HF/HSD (n = 12 each). (**B**) Daily ND intake by WT and KO mice during 12 and 16 weeks of age on ND (n = 10 each). (**C**) Daily HF/HSD intake by WT and KO mice during 12 and 16 weeks of age (n = 10 each). (**D**) Body lengths of WT and KO mice fed ND (12 and 16 weeks, n = 10 each). (**E**) Tissue weights of WT and KO mice fed ND (16 weeks, n = 8 each). Epi fat, epididymal fat. (**F**) Tissue weights of WT and KO mice fed HF/HSD (16 weeks, n = 8 each). (**G**) Body weights, excluding fat and viscera, of WT and KO mice fed ND or HF/HSD (16 weeks, n = 8 each). (**H**) Representative CT images of WT and KO mice fed HF/HSD at 16 weeks of age (yellow, fat; blue, lean tissue). (**I**) Body weight, (**J**) total fat, and (**K**) lean mass of WT and KO mice. Total fat and lean mass were calculated from CT scan data (16 weeks, n = 7 each). (**L**) Plasma leptin levels of WT and KO mice fed ND (10 weeks, n = 8 each). (**M**) Plasma leptin levels of WT and KO mice fed HF/HSD (16 weeks, n = 8 each). (**N**) Oxygen consumption (VO_2_) by WT and KO mice fed ND (16 weeks, n = 8 each). (**O**) VO_2_ of WT and KO mice fed HF/HSD (16 weeks, n = 8 each). (**P**) Heat production by WT and KO mice fed ND (16 week, n = 8 each). (**Q**) Heat production by WT and KO mice fed HF/HSD (16 weeks, n = 8 each). All data are expressed as the mean ± s.e.m. Data were analyzed by a two-way ANOVA (**A**), the Student’s *t*-test (**B–G, I–M**), or ANCOVA using body weight as a covariate (**N–Q**): *P < 0.05; **P < 0.01; ***P < 0.001; n.s., not significant.

*Ptprj*-KO mice are viable and fertile, and show no gross abnormalities^28^. However, we found that the body weights of and food intake by male *Ptprj*-KO mice on a normal diet (ND) were significantly lower than those of the corresponding WT littermates (Figs 1A,B and S1A). On a high-fat/high-sucrose diet (HF/HSD), the body weights of and food intake by *Ptprj*-KO mice were markedly lower than those of the corresponding WT littermates (Figs 1A,C and S1B). The body lengths of WT and *Ptprj*-KO mice were similar, suggesting no growth retardation in *Ptprj*-KO mice (Fig. 1D).

The epididymal fat pad weight of *Ptprj*-KO mice fed ND was significantly lower than that of their WT littermates, whereas no significant differences were observed in kidney, liver, spleen, or heart weights (Fig. 1E: 16 weeks old). *Ptprj*-KO mice fed HF/HSD exhibited lower epididymal fat pad, kidney, and liver weights than their WT littermates, while no significant differences were observed in spleen or heart weights between WT and KO mice (Fig. 1F: 16 weeks old). Body weights, excluding fat and viscera, were similar between WT and *Ptprj*-KO mice (Fig. 1G). Body composition analyzed by computed tomography (CT) scanning showed that fat mass mainly contributed to the difference in body weights between WT and *Ptprj*-KO mice (Fig. 1H–K). These results indicate that the lack of PTPRJ expression attenuated diet-induced obesity. Since male and female *Ptprj*-KO mice both showed a similar lean phonotype (Supplementary Fig. S2), male mice were used in subsequent experiments.

We examined plasma leptin levels. Plasma leptin levels were significantly lower in *Ptprj*-KO mice fed ND (10 weeks old) and HF/HSD (14 weeks old) than in their WT littermates (Fig. 1L,M). Since *Ptprj*-KO mice showed a lower food intake, notwithstanding their lower leptin levels, *Ptprj*-KO mice appeared to have higher sensitivity to leptin than WT mice.

Besides food intake, energy expenditure also affects body weight. Therefore, we evaluated the metabolic rate of *Ptprj*-KO mice by indirect calorimetry for a period of 96 h. No significant difference was noted in VO_2_ (Fig. 1N,O), heat production (Fig. 1P,Q), or VCO_2_ (Supplementary Fig. S1C,D) between WT and *Ptprj*-KO mice in the ND- and HF/HSD-fed groups. RER, which reflects the relative balance of carbohydrates and fat as a source of energy, was not significantly different between *Ptprj*-KO and WT mice fed ND (Supplementary Fig. S1E). However, the RER of *Ptprj*-KO mice fed HF/HSD was significantly higher than that of their WT littermates (Supplementary Fig. S1F), indicating that *Ptprj*-KO mice preferentially utilize carbohydrates over lipids. These results suggest that the lean phenotype of *Ptprj*-KO mice is mainly caused by a decreased food intake.

### PTPRJ is expressed in hypothalamic neurons

**Figure 2:**
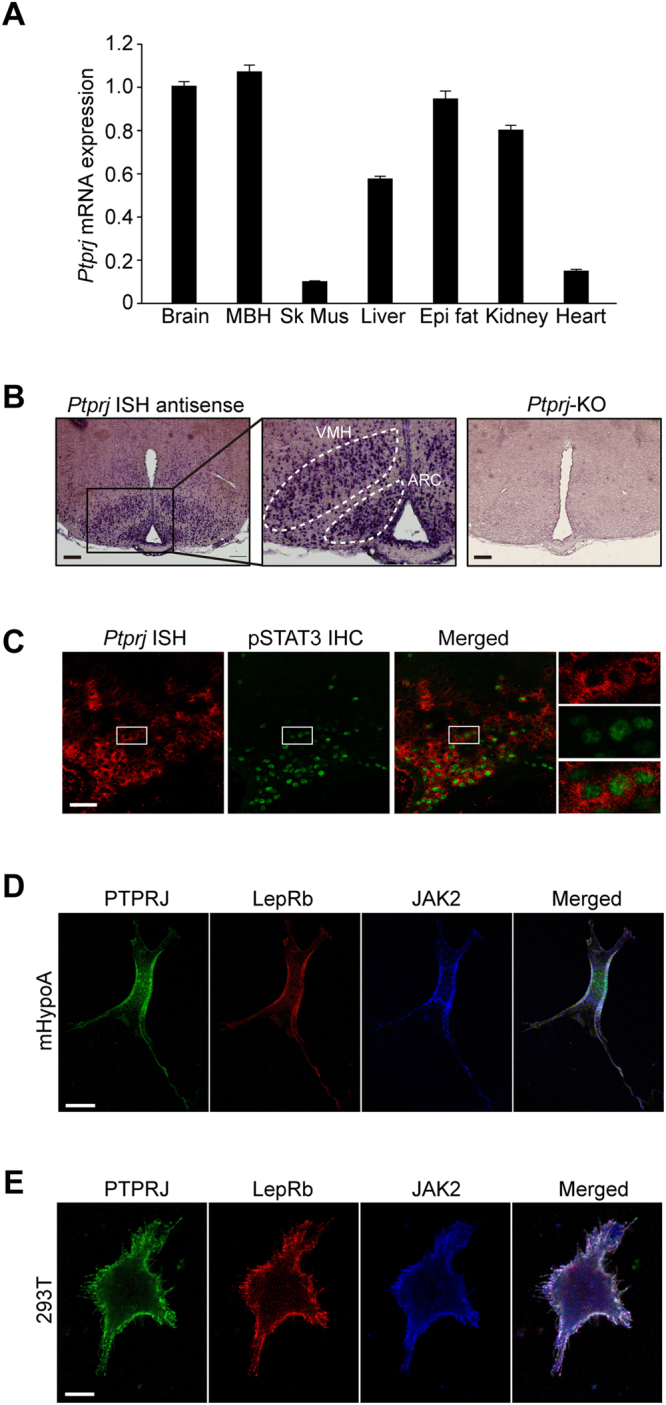
Expression of *Ptprj* in the hypothalamus. (**A**) Quantitative real-time RT-PCR analyses of *Ptprj* mRNA levels in various tissues (at 14 weeks of age, n = 4 each). Data are normalized to the expression level of *Gapdh*, and shown as relative values. Brain, whole brain; MBH, mediobasal hypothalamus; Sk Mus, skeletal muscle; Epi fat, epididymal fat. (**B**) *In situ* hybridization (ISH) of *Ptprj* mRNA in the mouse hypothalamus. The middle panel is a magnified view of the lined area in the left panel. The right panel is an ISH image of the *Ptprj*-KO mouse. ARC, arcuate nucleus; VMH, ventromedial hypothalamus. (**C**) Fluorescence *in situ* hybridization of *Ptprj* (red) and immunohistochemistry of leptin-induced pSTAT3 (green) in ARC. Brain specimens were prepared 30 min after the i.c.v. injection of leptin. Right-end panels are magnified views of the lined area in each panel. (**D**) Subcellular distributions of endogenous PTPRJ (green), LepRb (red), and JAK2 (blue) in the immortalized murine neuronal cell line, mHypoA-2/10. (**E**) Subcellular distributions of PTPRJ (green), LepRb (red), and JAK2 (blue) exogenously expressed in HEK293T cells. Cells were treated with 50 ng/ml leptin for 15 min, and fixed for immunostaining in (**D**) and (**E**). Scale bars: (**B**) 200 μm, (**C**) 50 μm, (**D**,**E**) 10 μm.

Leptin acts on ARC in the hypothalamus to regulate food intake and energy expenditure^6^. Therefore, we investigated whether PTPRJ is expressed in the hypothalamus. The strong expression of *Ptprj* mRNA in the mediobasal hypothalamus (MBH) was demonstrated in a quantitative RT-PCR analysis (Fig. 2A). We confirmed the protein expression of PTPRJ in MBH by Western blotting (Supplementary Fig. S3). *In situ* hybridization (ISH) analyses also revealed that *Ptprj* mRNA was expressed in the MBH, especially in ARC and the ventromedial hypothalamus (VMH) (Fig. 2B). A combination of ISH for *Ptprj* and immunohistochemistry (IHC) for phosphorylated STAT3 (pSTAT3) in ARC showed that pSTAT3 signals in the nuclei were surrounded by *Ptprj* signals in the cytoplasms after intracerebroventricular (i.c.v.) administration of leptin (Fig. 2C). Activated STAT3 (pSTAT3) is known to appear in the nuclei of cells in response to leptin, therefore, it would be reasonable to conclude that PTPRJ is expressed in leptin-sensitive ARC neurons in the MBH.

We further examined the subcellular distribution of PTPRJ, LepRb, and JAK2 in the immortalized murine hypothalamic cells line, mHypoA-2/10 (ref. 29). Immunocytochemistry indicated that PTPRJ proteins co-localized well with LepRb and JAK2 at the cell surface not only before (data not shown) but also after the leptin stimulation in mHypoA-2/10 cells (Fig. 2D). This co-localization was confirmed in HEK293T cells in which the expression of these three molecules was artificially induced (Fig. 2E). Taken together with the phenotype of *Ptprj*-KO mice, these results strongly suggested that PTPRJ regulates leptin signaling in the hypothalamus.

### PTPRJ suppresses leptin signaling through the dephosphorylation of JAK2 at Y813 and Y868

**Figure 3:**
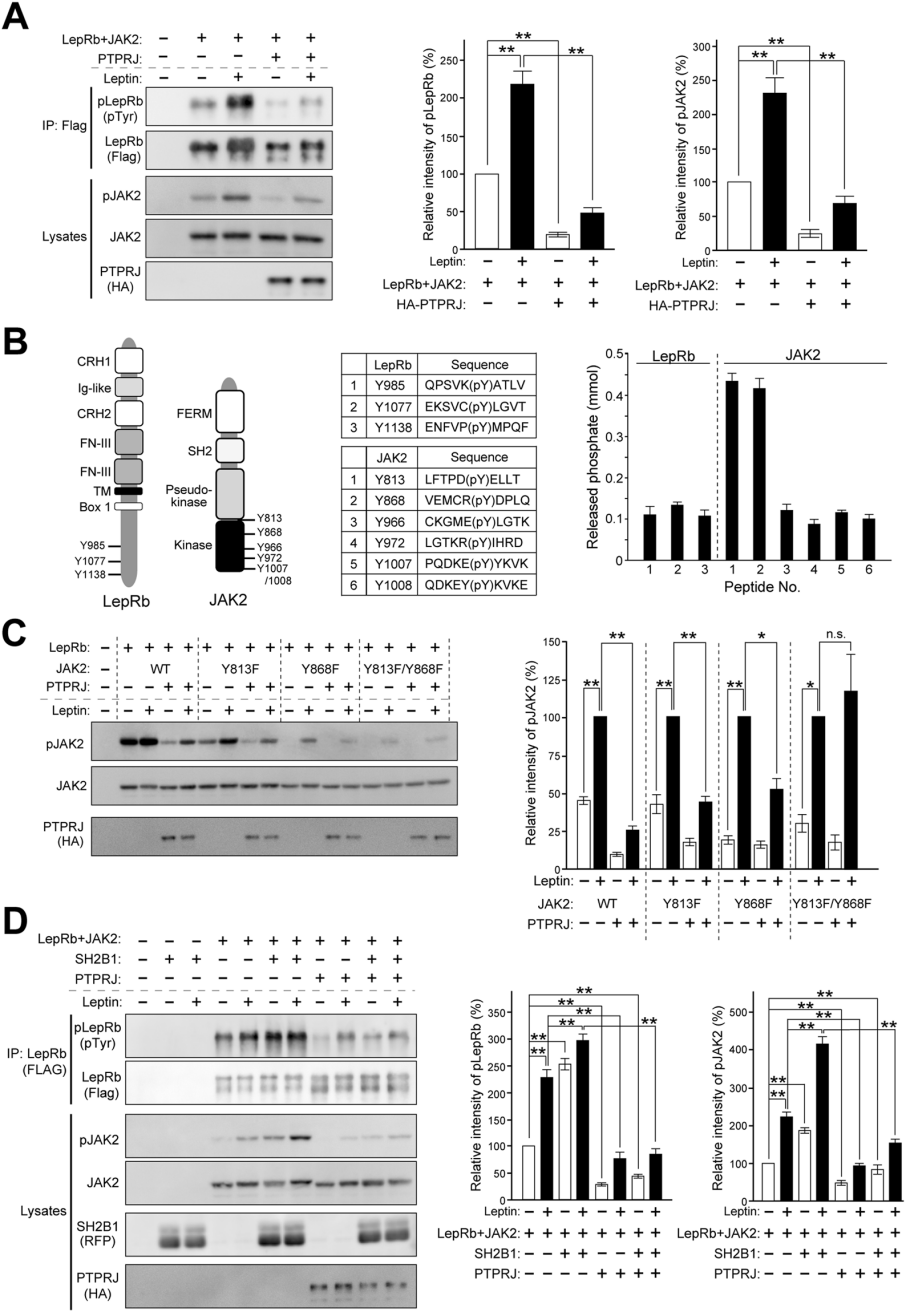
PTPRJ suppresses leptin signaling through the dephosphorylation of Y813 and Y868 in JAK2. (**A**) Suppression of the tyrosyl phosphorylation of LepRb and JAK2 by the co-expression of PTPRJ in HEK293T cells. FLAG-LepRb and JAK2 expression constructs were co-transfected with an empty vector or HA-PTPRJ expression construct. Cells were treated with/without 50 ng/ml leptin for 15 min, and LepRb proteins were immunoprecipitated with an anti-FLAG antibody and analyzed with the indicated antibodies. The expression and phosphorylation of JAK2 in lysates were analyzed with anti-JAK2 and phospho-JAK2 (pY1007/1008) antibodies. PTPRJ proteins were detected with an anti-HA antibody. The right two graphs show summaries of the phosphorylation levels of LepRb and JAK2 (n = 4). Full-length blots are presented in Supplementary Fig. S9. (**B**) Dephosphorylation assays using synthetic phosphopeptides. Schematic representation of phosphorylation sites in LepRb and JAK2 (left). CRH1; cytokine receptor homology 1: CRH2; cytokine receptor homology 2: Ig-like; immunoglobulin-like: FN-III, fibronectin type III repeat: Box 1; box 1 motif: FERM; four-point-one, ezrin, radixin, moesin domain: SH2; src homology 2. List of phosphopeptides (middle). Summary of phosphatase assays (right). Phosphopeptides were incubated with GST-PTPRJ, and the amount of phosphate released was measured (n = 3). (**C**) Tyrosine phosphorylation levels of wild-type and mutant JAK2 with or without PTPRJ expression. The right graph shows a summary of the phosphorylation levels of JAK2 (n = 4). Full-length blots are presented in Supplementary Fig. S10. (**D**) Suppression of the SH2B1-enhanced phosphorylation of LepRb and JAK2 by the co-expression of PTPRJ in HEK293T cells. An mCherry-SH2B1 expression construct was co-transfected with the indicated constructs. The right two graphs show summaries of the phosphorylation levels of LepRb and JAK2 (n = 4 each). Full-length blots are presented in Supplementary Fig. S11. The graphs of (**A)** and (**D**) show relative values to that without the leptin stimulation, and the graph of (**C**) shows relative values to that with the leptin stimulation. All data are expressed as the mean ± s.e.m. *P < 0.05 and **P < 0.01 represent significant differences between the indicated pairs (ANOVA followed by Scheff’s post-hoc test).

In order to elucidate the regulatory mechanisms underlying leptin signaling by PTPRJ, we investigated whether PTPRJ affects LepRb/JAK2 activation by leptin in HEK293T cells. JAK2 activity was monitored by the phosphorylation of Tyr1007 (Y1007) and Y1008 in JAK2, critical tyrosines in the activation loop of JAK2. When HEK293T cells expressing LepRb and JAK2 were stimulated with leptin, both of these proteins became highly tyrosine-phosphorylated (Fig. 3A). When PTPRJ was co-expressed, the tyrosine phosphorylation levels of LepRb and JAK2 were strongly suppressed before and after the leptin stimulation (Fig. 3A). These results indicate that PTPRJ is a negative regulator of leptin signaling.

We then attempted to identify tyrosine residues in LepRb and JAK2 that are dephosphorylated by PTPRJ. After the leptin stimulation, specific tyrosine residues are reportedly phosphorylated in LepRb and JAK2 (Fig. 3B, left). The major phosphorylation sites in LepRb are Y985, Y1077, and Y1138 (ref. 30). In addition, JAK2 activity is known to be positively regulated by phosphorylation at Y813, Y868, Y966, Y972, Y1007, and Y1008 (refs 31 and 32). We initially performed *in vitro* phosphatase assays using synthetic phosphopeptides containing these phosphorylated tyrosine residues in the mouse LepRb and JAK2 sequences individually (Fig. 3B, middle). PTPRJ preferentially dephosphorylated the two peptides containing phosphorylated Y813 and Y868 of the JAK2 sequence, but none of the LepRb sequence (Fig. 3B, right).

In order to confirm the specificity of dephosphorylation sites by PTPRJ and the importance of dephosphorylation at Y813 and Y868 in JAK2, we prepared the following JAK2 mutants: JAK2(Y813F), JAK2(Y868F), and JAK2(Y813/868F), in which the corresponding tyrosine (Y) was replaced with phenylalanine (F); Phenylalanine mimics the dephosphorylated tyrosine residue. These JAK2 mutants and LepRb were expressed together with or without PTPRJ in HEK293T cells, and JAK2 activation levels were examined by monitoring the tyrosine phosphorylation of Y1007/1008 in JAK2.

The basal and leptin-stimulated tyrosine phosphorylation levels of JAK2(Y813F) were both significantly lower than those of wild-type JAK2 (Fig. 3C). The mutation at Y868 resulted in a more prominent reduction in the tyrosine phosphorylation of JAK2 (Fig. 3C), as previously reported^31^. Moreover, JAK2 double mutant Y813F/Y868F proteins (JAK2(Y813/868F)) were scarcely phosphorylated (activated) by leptin (Fig. 3C). These results suggest that the simultaneous phosphorylation of Y813 and Y868 plays a pivotal role in JAK2 activation by leptin.

When PTPRJ was co-expressed with wild-type JAK2, JAK2(Y813F), or JAK2(Y868F), the tyrosine phosphorylation levels of these mutants were significantly reduced before and after the leptin stimulation (Fig. 3C). However, PTPRJ co-expression did not suppress the leptin-induced phosphorylation of JAK2(Y813/868F) anymore (Fig. 3C). These results support PTPRJ preferentially dephosphorylating Y813 and Y868 in JAK2.

### PTPRJ suppresses leptin signaling enhanced by SH2B1

The autophosphorylation of Y813 in JAK2 reportedly serves as a binding site for SH2B1, an SH2 domain-containing adaptor protein expressed in the central nervous system and peripheral tissues^32–34^. SH2B1 enhances leptin signaling through the augmentation of JAK2 activity, and deletions or mutations in the *SH2B1* gene are known to be associated with severe obesity in humans and mice^35–37^. Thus, we examined the effects of PTPRJ on the interaction between JAK2 and SH2B1, and SH2B1 activity in leptin signaling.

SH2B1 constitutively binds to JAK2 via the N-terminal region of SH2B1, and a leptin stimulation promotes their interaction through the phosphorylation of Y813 in JAK2 (ref. 33). As previously reported, when SH2B1 was co-expressed with JAK2 and LepRb, the leptin stimulation enhanced the interaction between JAK2 and SH2B1 (Supplementary Fig. S4 **)**. However, when PTPRJ was additionally co-expressed, the amount of co-immunoprecipitated SH2B1 with JAK2 was significantly reduced before and after the leptin stimulation (Supplementary Fig. S4 **)**.

We also examined the effects of PTPRJ expression on leptin signaling enhanced by SH2B1. As reported previously, when SH2B1 was co-expressed with LepRb and JAK2 in HEK293T cells, the tyrosine phosphorylation levels of LepRb and JAK2 proteins were significantly enhanced with and without the leptin stimulation (Fig. 3D). However, the additional co-expression of PTPRJ strongly suppressed the SH2B1-enhanced tyrosine phosphorylation of LepRb and JAK2 (Fig. 3D). Collectively, these results indicate that PTPRJ suppresses SH2B1-dependent enhancements in leptin signaling by inhibiting the interaction between JAK2 and SH2B1 via the dephosphorylation of Y813 in JAK2.

### The hypothalamus of *Ptprj-*KO mice show enhanced leptin sensitivity

We then examined whether PTPRJ regulates food intake through the inhibition of leptin signaling in the hypothalamus. The overall brain structure and tissue organization in *Ptprj*-KO mice had a normal appearance, as assessed by HE staining (data not shown). An immunohistochemical analysis of the distribution of neurons expressing NPY or the hypothalamic POMC-derived peptide, α-melanocyte-stimulating hormone (α-MSH), showed no marked difference in the hypothalamus between *Ptprj*-KO and WT mice (Supplementary Fig. S5A,B). Furthermore, no significant difference was observed in the expression levels of LepRb, JAK2, SH2B1, STAT3, and SOCS3 in MBH between *Ptprj*-KO and WT mice (Supplementary Fig. S3).

**Figure 4:**
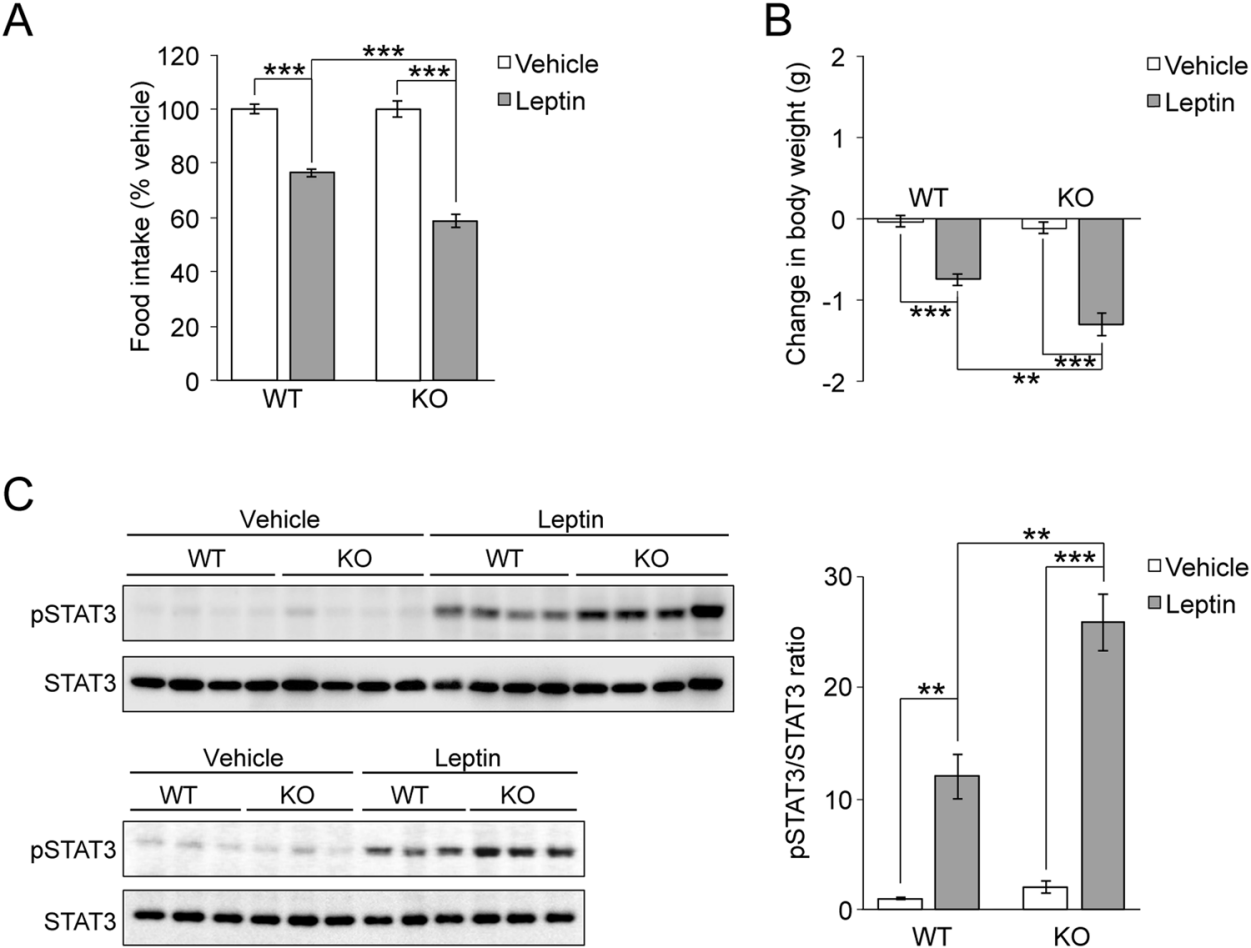
*Ptprj*-KO mice show enhanced central leptin sensitivity. (**A**) Changes in food intake and (**B**) Changes in body weight during 24 h by the i.c.v. administration of leptin. Leptin (2 μg) or vehicle was injected 1 h before the onset of the dark phase in WT and KO mice. Mice were fed ND (13 weeks of age, n = 10 each). (**C**) Western blot analyses of pSTAT3 and total STAT3 in the MBH lysates prepared from WT and KO mice (n = 7 each). Mice were fasted for 18 h, injected with leptin (2 μg) or vehicle, and killed 30 min later. The left panels show Western blotting image. Data are individually composed of different mouse samples. The right graph shows a summary of quantitative analyses, which represents the relative ratio of pSTAT3/STAT3. Full-length blots are presented in Supplementary Fig. S12. Data are expressed as the mean ± s.e.m. **P < 0.01, ***P < 0.001 represents significant differences between the indicated pairs (ANOVA followed by Scheffe’s post-hoc test).

After the i.c.v. injection of leptin, food intake and body weight were significantly reduced in WT and *Ptprj*-KO mice (Fig. 4A,B). Of note, the degree of this reduction was greater in *Ptprj*-KO mice (Fig. 4A,B). We examined pSTAT3 levels in the MBH as a readout of LepRb activation. pSTAT3 levels (pSTAT3/total STAT3 ratio) in the MBH of WT and *Ptprj*-KO mice were increased ~13-fold upon the leptin treatment (Fig. 4C). Importantly, *Ptprj*-KO mice exhibited a ~2-fold stronger activation of STAT3 than WT mice (Fig. 4C; 25.2 ± 3.3 and 12.8 ± 3.1), indicating their enhanced leptin sensitivity.

### PTPRJ contributes to the development of leptin resistance

**Figure 5:**
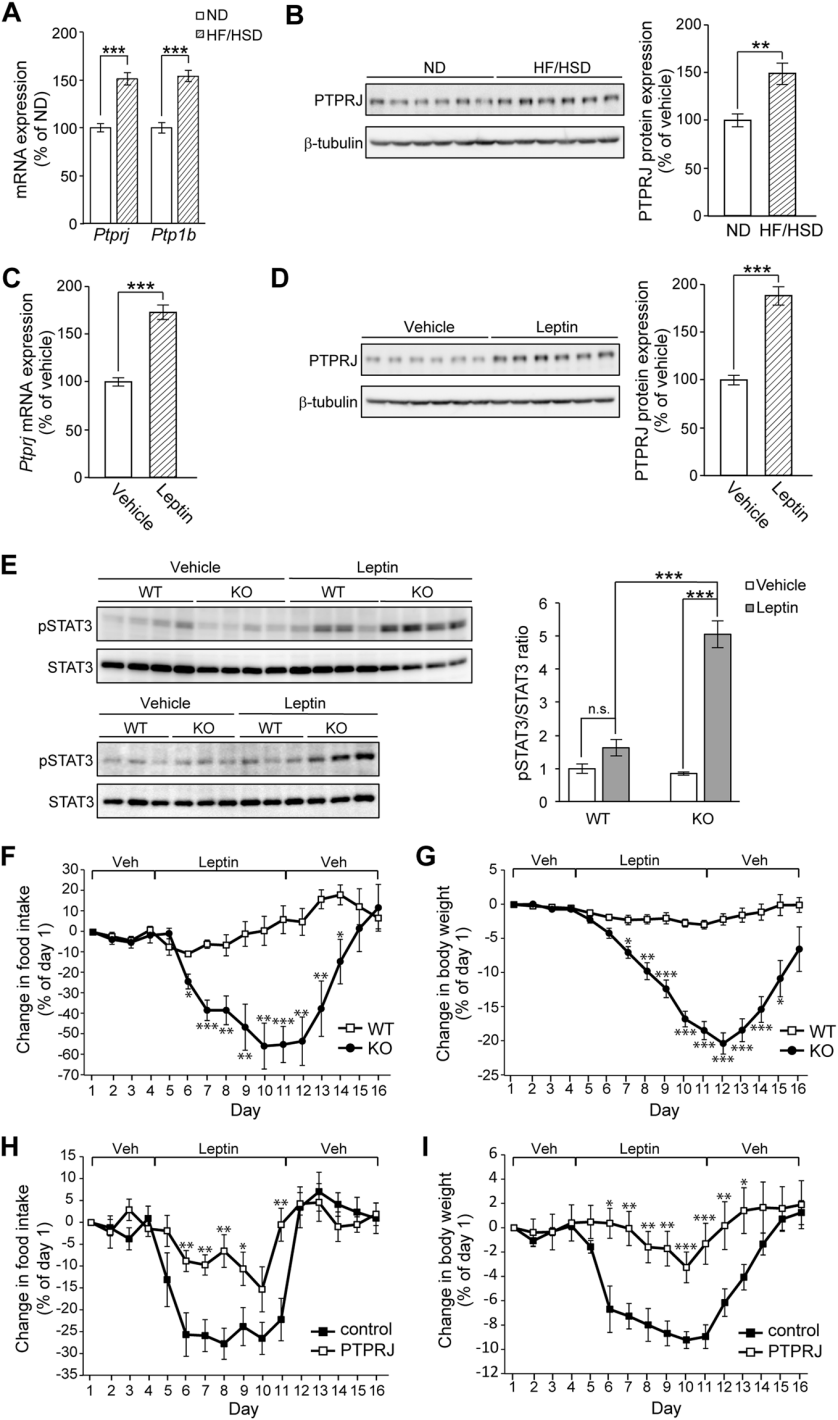
PTPRJ contributes to the development of leptin resistance. (**A**) Quantitative real-time RT-PCR analyses of *Ptprj* and *Ptp1b* mRNA levels in the MBH of WT mice fed ND or HF/HSD for 8 weeks (at 14 weeks of age, n = 6 each). The expression levels of mRNA were normalized to the *Gapdh* mRNA content, respectively. (**B**) Western blot analyses of PTPRJ and β-tubulin in MBH lysates from mice fed ND or HF/HSD for 8 weeks (at 14 weeks of age, n = 6). The sample of each lane represents an analysis of an individual mouse. The right graph shows a summary of quantitative analyses. (**C**) Quantitative real-time RT-PCR analyses of *Ptprj* mRNA levels and (**D**) Western blot analyses of PTPRJ and β-tubulin in the MBH of *ob*/*ob* mice treated with leptin (2 μg) or vehicle for 3 days (n = 6 each). (**E**) Western blot analyses of pSTAT3 and total STAT3 in the MBH lysates of WT and KO mice fed HF/HSD for 14 weeks (at 18 weeks of age, n = 7 each). Mice were fasted for 18 h, injected with leptin (2 μg) or vehicle, and killed 30 min later. (**F**) Daily change in food intake by and **(G)** Daily change in body weights of WT and KO mice fed HF/HSD for 14 weeks upon daily administration of leptin (n = 8 each). Leptin (500 ng) or vehicle was i.c.v. injected as indicated in WT and KO mice. (**H**) Daily change in food intake by and (**I**) Daily change in body weights of lean mice infected with AAV-GFP (control) or AAV-PTPRJ (PTPRJ) upon the i.c.v. injection of leptin. Mice fed ND were used at 12–16 weeks of age (n = 8 each). Experiments were performed as indicated in (**F**). All data are expressed as the mean ± s.e.m. The Student’s *t*-test (**A–D**) and ANOVA (**E–I**) were used to detect significant differences between the indicated groups (*P < 0.05, **P < 0.01, ***P < 0.001). n.s., not significant. Full-length blots of (**B**), (**D**), and (**E**) are presented in Supplementary Fig. S13.

The induction of PTP1B, a negative regulator of LepRb signaling in the hypothalamus, has been reported as a possible cause of leptin resistance^20^. Leptin resistance is induced by the feeding of HF/HSD along with obesity. Therefore, we assessed PTPRJ expression levels in MBH in HF/HSD-fed mice and ND-fed mice. HF/HSD feeding for 8 weeks significantly increased the *Ptp1b* mRNA level in MBH (Fig. 5A; see also ref. 20). This is also the case for the *Ptprj* mRNA (Fig. 5A). Consistently, the expression levels of PTPRJ proteins in HF/HSD-fed mice showed a similar increase (~1.5-fold) (Fig. 5B).

Leptin signaling is known to regulate the expression of several genes implicated in the regulation of energy homeostasis^12^. We examined the effects of leptin on *Ptprj* expression in MBH using leptin-deficient *ob*/*ob* mice. The mRNA and protein levels of PTPRJ were markedly increased by the i.c.v. injection of leptin (~1.7 and ~1.8 fold vs. vehicle, respectively) (Fig. 5C,D), suggesting that *Ptprj* gene expression in MBH is regulated by leptin.

We then examined the induction of leptin resistance in *Ptprj*-KO mice fed HF/HSD for 14 weeks. Leptin signaling was evaluated by assessing pSTAT3 levels after *Ptprj*-KO and WT mice were treated with leptin or vehicle. Leptin administration to WT mice only had a small effect on the phosphorylation of STAT3 (Fig. 5E), indicating attenuated leptin signaling in these mice. In contrast, pSTAT3 levels in the MBH of *Ptprj*-KO mice were markedly higher after leptin injection than those in vehicle-treated mice (Fig. 5E), indicating that the lack of PTPRJ expression potently attenuated the development of leptin resistance. When we compared the effects of ND and HF/HSD feeding on the leptin-induced phosphorylation of STAT3, the levels of pSTAT3 in *Ptprj*-KO mice fed HF/HSD were significantly increased upon leptin administration, but evidently lower than those in ND-fed KO mice (Supplementary Fig. S6).

In order to examine the effects of the leptin treatment on body weight loss, leptin was i.c.v. injected for 7 days into WT and *Ptprj*-KO mice fed HF/HSD. WT mice exhibited strong leptin resistance; i.c.v. leptin administration only exerted a slight reduction in food intake and body weight (Fig. 5F,G; WT). In contrast, leptin administration strongly and continuously reduced food intake and body weight in *Ptprj*-KO mice during its administration (Fig. 5F,G; KO). The effects of leptin were rapidly reversed within 48 h after its administration had ceased. These results indicated that a deficiency in PTPRJ strongly attenuated the development of diet-induced leptin resistance.

We then investigated the effects of the overexpression of PTPRJ in ARC on leptin resistance in lean mice. We injected an adeno-associated virus (AAV) carrying the gene encoding PTPRJ into the ventral hypothalamus (Supplementary Fig. S7), and examined the effects of leptin administration on body weight loss. When leptin was i.c.v. injected for 7 days, marked reductions in food intake and body weight were observed in ND-fed male WT mice with the AAV-mediated expression of GFP (control mice) (Fig. 5H,I). These leptin effects rapidly disappeared within 24 h of the cessation of leptin administration. In contrast, mice with the overexpression of PTPRJ in ARC showed small decreases in food intake and body weight during leptin administration (Fig. 5H,I). These results indicate that the overexpression of PTPRJ in MBH induces leptin resistance in lean mice.

## Discussion

We previously demonstrated that PTPRJ is involved in the regulation of insulin signaling by attenuating activation of the IR^26^. In the present study, we revealed that PTPRJ is also involved in the regulation of leptin signaling through the dephosphorylation of JAK2, the primary tyrosine kinase in leptin signaling: PTPRJ attenuates leptin signaling by dephosphorylating Y813 and Y868 in JAK2. Biochemical experiments using JAK2 mutants revealed that the simultaneous phosphorylation of Y813 and Y868 plays a pivotal role in JAK2 activation. In line with these results, deletion of the *Ptprj* gene in mice resulted in enhanced leptin sensitivity in the hypothalamus, and *Ptprj*-KO mice exhibited lower food intake and leaner phenotypes than WT mice. When fed HF/HSD, *Ptprj*-KO mice also gradually got obese due to a decrease in leptin sensitivity as judged by the activation of STAT3, but they exhibited attenuated development of leptin resistance with obesity. Upon leptin administration, *Ptprj*-KO mice fed HF/HSD showed significant reductions in food intake and body weight as compared with corresponding WT mice. Moreover, the overexpression of PTPRJ in MBH in non-obese mice with a viral vector induced significant leptin resistance. Overall, our results indicated that PTPRJ plays critical roles in the development of leptin resistance *in vivo*.

**Figure 6:**
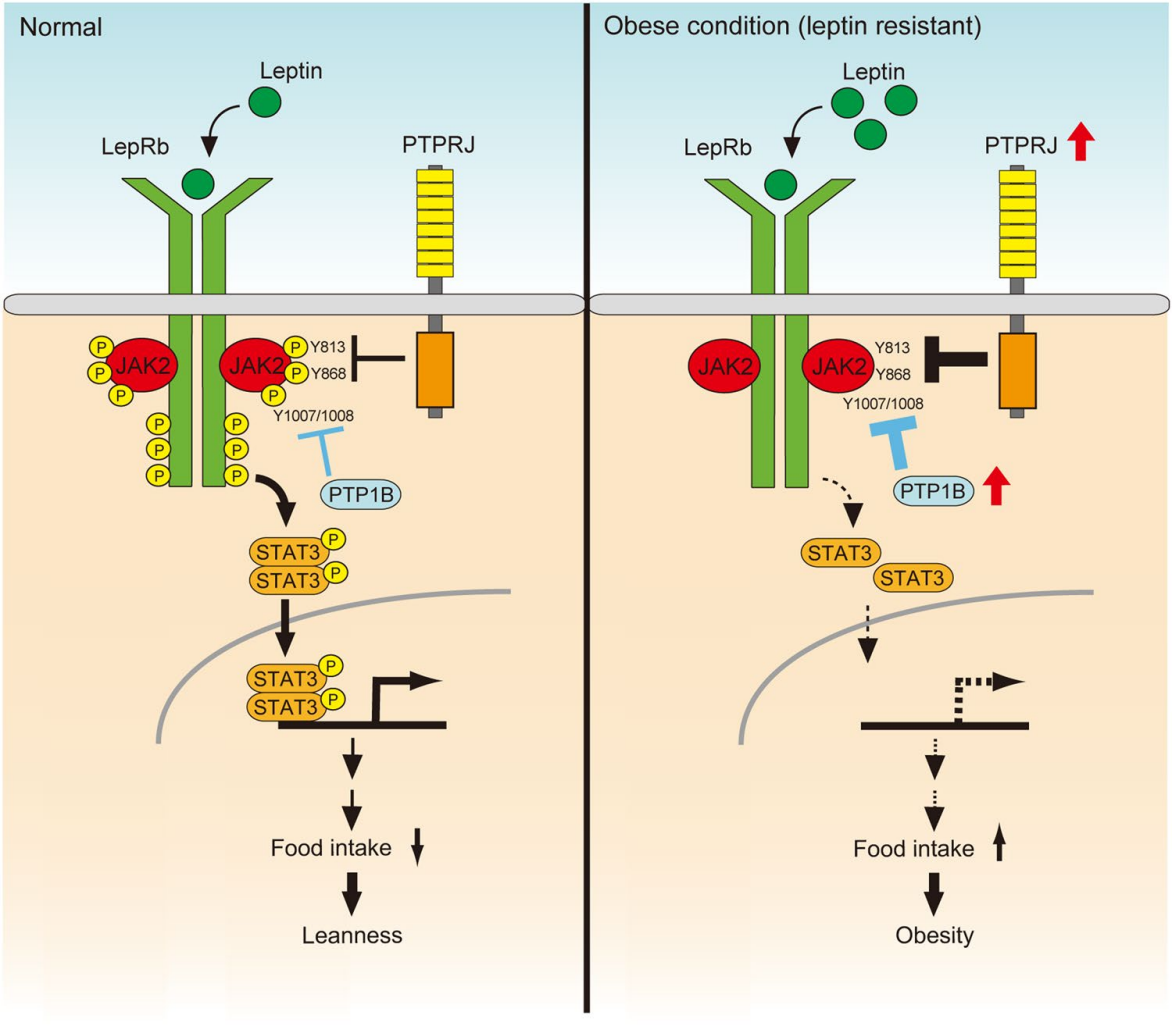
Schematic drawings representing the proposed role of PTPRJ in leptin signaling. PTPRJ negatively regulates leptin signaling by inhibiting JAK2 activation. In diet-induced obesity, PTPRJ expression is up-regulated in the hypothalamus, which may contribute to the development of leptin resistance.

Obesity is known to lead to leptin resistance, which subsequently exacerbates obesity and hyperphagia^30^. The suppression of leptin signaling by the increased expression of negative regulators in the hypothalamus is considered to be one of the principal mechanisms of leptin resistance^30,38^. PTP1B and SOCS3 are well-known negative regulators of leptin signaling, which inhibit leptin signaling by dephosphorylating JAK2 and binding to phosphorylated LepRb, respectively^13,14,39,40^. Moreover, PTP1B and SOCS3 contribute to the development of leptin resistance: Their expression in the hypothalamus is increased by diet-induced obesity, and their deficiency protects mice from the development of leptin resistance^41,42^. We showed that *Ptprj* is expressed in leptin-sensitive ARC neurons (Fig. 2C). This notion is consistent with the recent finding by Campbell *et al*., that substantial levels of *Ptprj* mRNA are expressed in LepR-positive POMC and AgRP neurons in the ARC^43^. The expression of *Ptprj* in MBH was up-regulated in response to both HF/HSD intake and leptin administration. Because *Ptprj* expression is null in *Ptprj*-KO mice, they were substantially protected from the development of leptin resistance (Fig. 6).

Some PTPs, including PTP1B, T-cell protein tyrosine phosphatase (TCPTP), and protein tyrosine phosphatase receptor type E (PTPRE), have been reported to regulate leptin signaling^39,41,44–46^. Among them, PTP1B has been studied well from the viewpoint of a therapeutic target for obesity^47,48^. As with *Ptp1b*-deficient mice^40,41^, we herein demonstrated that *Ptprj*-deficient mice showed leptin hypersensitivity. The reduced food intake and body weight gain by *Ptprj*-deficient mice are similar to those by *Ptp1b*-deficient mice (Fig. 1 and refs 40 and 41). Additionally, both mice exhibited the attenuation of diet-induced leptin resistance (Figs 4 and 5 and refs 40–42).

Upon the binding of leptin to LepRb, JAK2 is activated by autophosphorylation at specific tyrosine residues. Individual tyrosine phosphorylation sites in JAK2 have been functionally characterized: The phosphorylation of Y1007/Y1008 in the activation loop is critical for JAK2 activation, while that of Y813, Y868, Y966, and Y972 has been shown to potentiate JAK2 activity^31,32^. PTPRJ and PTP1B have different dephosphorylation site specificities in JAK2: PTPRJ preferentially dephosphorylates Y813 and Y868 (Fig. 3B,C), while PTP1B reportedly dephosphorylates Y1007/Y1008 in the activation loop of JAK2 (ref. 49). PTPRJ and PTP1B also show different specificities for dephosphorylation sites in the IR: In the mouse IR, PTPRJ preferentially dephosphorylates Y960 and Y1146 (ref. 26), whereas PTP1B dephosphorylates Y1150/Y1151 in the activation loop^50^. Consistent with distinct specificities in dephosphorylation sites, the subcellular distribution of PTPRJ and PTPP1B differ: PTPRJ is distributed in the plasma membrane, whereas PTP1B is localized at the ER via its C-terminal segment^51^. Thus, PTPRJ and PTP1B appear to regulate leptin and insulin signaling in different manners. As long as we assessed by literature searches, we could not identify any marked differences in phenotypes between *Ptp1b*- and *Ptprj*-deficient mice.

LepRb is widely expressed throughout the body, and leptin exerts its functions on peripheral tissues as well as the central nervous system^52^. *Ptprj* and *Ptp1b* mRNAs showed similar distributions in the brain, but different in peripheral tissues: *Ptprj* expression levels were high in the liver, fat, and kidney, but low in the skeletal muscle and heart (Fig. 2A), while *Ptp1b* expression levels were high in the skeletal muscle, fat, kidney, and heart, but low in the liver (Supplementary Fig. S8). These results suggest that PTPRJ mainly regulates signaling in the liver, adipocytes, and kidney in peripheral tissues. The expression of *Ptprj*, but not *Ptp1b* was specifically increased in the liver by feeding HF/HSD only for 2 weeks (our unpublished observations), indicating that PTPRJ plays a major role in regulating leptin and insulin signaling in the liver and brain. In line with this view, the muscle-specific deletion of *Ptp1b* resulted in hyperphosphorylation of the muscle IR without an insulin stimulation, whereas the liver-specific deletion of *Ptp1b* did not exerted almost no effect to liver IR^53,54^.

Among tissue-specific *Ptp1b*-deficient mice, only neuron-specific deficient mice exhibited reduced food intake and lower body weights, and they were specifically protected from developing leptin resistance^55^. In addition, neuron-specific *Socs3*-deficient mice showed reduced food intake and lower body weights as well as resistance to diet-induced obesity^14^. Therefore, we speculate that reduced food intake by *Ptprj*-deficient mice is attributable to the lack of PTPRJ in the brain. The adipocyte-specific deletion of PTP1B resulted in an increased body weight^55^ and adipocyte-specific *IR*-deficient mice are protected from obesity^56^; however, it is difficult at present stage to estimate the extent of the contribution of enhanced insulin signaling in peripheral tissues to the lean phenotype of *Ptprj*-deficient mice. No significant difference was observed in energy expenditure between WT and *Ptprj*-deficient mice. The deletion of *Ptprj* in peripheral tissues may attenuate the effects of up-regulated leptin signaling in the hypothalamus on energy expenditure in *Ptprj*-deficient mice. In order to clarify the contribution of each tissue to the effects of PTPRJ on the regulation of food intake and energy expenditure, tissue-specific *Ptprj*-deficient mice need to be generated and analyzed in the future.

Phosphorylated Y813 is known to serve as a binding site for SH2B1 (refs 32 and 33). SH2B1 is an activator of leptin signaling, and deletions or mutations in the *SH2B1* gene are associated with severe obesity in humans and mice^35–37^. We herein showed that PTPRJ suppressed SH2B1-dependent enhancements in leptin signaling by inhibiting the interaction between JAK2 and SH2B1 via the dephosphorylation of Y813 in JAK2. Consistent with these results, *Ptprj*- and *Sh2b1*-deficient mice showed contrasting phenotypes: *Ptprj*-deficient mice showed enhanced leptin signaling and a lean phenotype, while *Sh2b1*-deficient mice showed impaired leptin signaling and severe obesity. Therefore, PTPRJ may be a negative regulator of SH2B1. SH2B1 is also known to enhance insulin signaling^34,57^. In line with this view, PTPRJ dephosphorylates Y1146 in the activation loop of the mouse IR, which is also the binding site of SH2B1 (ref. 58). Thus, PTPRJ and SH2B1 have counteracting functions in insulin and leptin signaling.

In summary, *Ptprj*-KO mice exhibit improved glucose and insulin tolerance through enhanced insulin signaling^26^. We herein demonstrated that *Ptprj*-KO mice exhibited lean phenotypes with lower adiposity because of reduced food intake through enhanced leptin signaling. Moreover, we found that the induction of PTPRJ may contribute to leptin resistance induced by obesity. These results indicate that PTPRJ serves as a novel potential therapeutic target for the treatment of obesity and type 2 diabetes.

## Methods

### Ethics statement and experimental animals

All procedures were approved by the Institutional Animal Care and Use Committee of the National Institutes of Natural Sciences, Japan, and were performed in accordance with the guidelines of the Institutional Committee for the Use of Animals for Research. C57BL/6J and C57BL/6-Lepob/J (*ob*/*ob*) mice were purchased from Clea Japan and Charles River Japan, respectively. *Ptprj* heterozygous mice^29^ were backcrossed to a C57BL/6 background to give a final seven-generation C57BL/6 congenic. After the final backcross, *Ptprj* ^+/−^ mice were interbred to give littermates for analyses. Mice were housed under a constant room temperature (23 °C) and 50–55% humidity in specific pathogen-free conditions on a 8:00 to 20:00 light cycle. All sex-matched littermates were housed in plastic cages (cage size: 12 × 21 × 12.5 cm) containing wood shavings. Surgeries for paraformaldehyde fixation, i.c.v. cannulation, and virus injection were performed under pentobarbital-xylazine anesthesia, and all efforts were made to minimize suffering.

### Food intake, body weight, and tissue weight

Mice were fed chow diet (CA-1, Clea Japan) or a high-fat/high-sucrose diet (F2HFHSD, Oriental Yeast) consisting of protein (17.2% of energy intake), fat (54.5%), and carbohydrate (28.3%). Body weight and food intake were measured daily at 19:00 to 20:00 pm. The tissues were dissected and weighed.

### Computed tomography (CT) scanning

CT scanning was performed using an experimental CT system (LaTheta LCT-200; Hitachi-Aloka Medical) under isoflurane anesthesia. Mice were scanned at a resolution of 192 μm. An overview scanning image of the whole mouse was created to allow the selection of regions of interest for subsequent scans. Fat and muscle contents in the body were calculated from images between cervical 2 and base of the tail using LaTheta software.

### Plasma leptin

Blood samples were collected from the tail vein between 10:00 and 11:00 am. Plasma was separated by centrifugation (3,000 × *g*) for 10 min, and stored at −80 °C until the time of the leptin assay. Plasma leptin was measured using a Mouse/Rat Leptin ELISA kit (Morinaga).

### Indirect calorimetry

Indirect calorimetry was performed using the Oxymax system (Columbus Instruments’ Comprehensive Lab Animal Monitoring System (CLAMS)). Mice were placed in individual cages with a 12:12-h light/dark cycle. After a 16-h adaptation, the system measured O_2_ consumption (VO_2_), CO_2_ production (VCO_2_), heat production, and the respiratory exchange ratio (RER) for each mouse for 96 h. Measurements of O_2_ and CO_2_ were taken every 10 min, and the values of the light and dark phase were averaged. Data were analyzed by ANCOVA using body weight as a covariate^59^.

### Antibodies

Anti-FLAG (SIG1–25), anti-HA (HA-7), and anti-β-tubulin (SDL.3D10) antibodies were purchased from Sigma-Aldrich. The anti-RFP antibody was from Thermo Fisher Scientific. Anti-phosphotyrosine (4G10) and anti-NeuN (A60) antibodies were from Merck Millipore. The anti-PTPRJ (AF1934) antibody was from R&D Systems. Anti-SH2B1 (E-20) and anti-OB-R (B-3) antibodies were from Santa Cruz Biotechnology. Anti-JAK2 (D2E12), anti-STAT3 (124H6), anti-phosphorylated STAT3 (phospho Y805, D3A7), and anti-SOCS3 (L210) antibodies were from Cell Signaling Technologies. The anti-phosphorylated JAK2 (phospho Y1007 + Y1008, E132) antibody was from Abcam. Anti-NPY (22940) and anti-α-MSH (20074) antibodies were from Immunostar. HRP-conjugated and Alexa-conjugated secondary antibodies were from Thermo Fisher Scientific.

### *In situ* hybridization

Mice were fixed with 4% paraformaldehyde (PFA) in 0.1 M phosphate buffer (pH 7.4), and then brains were removed. The *in situ* hybridization of hypothalamic sections (thickness of 18 μm) was performed as previously described^26^.

### Fluorescence *in situ* hybridization and immunohistochemistry

Tissue sections were reacted with the DIG-AP antibody and primary antibody after hybridization using the above procedure. After washes in TBS, tissue sections were incubated with Alexa Flour 488. After washes in TBS, sections were reacted with Fastred (Sigma-Aldrich) according to the manufacturer’s instructions. Double-stained sections were mounted and examined using the LSM-700 confocal laser microscope (Zeiss).

### DNA constructs

Mouse LepRb, JAK2, and SH2B1 cDNAs were cloned by RT-PCR using total RNA from the mouse brain using the following primers. LepRb: forward, 5′-AAGCTTCCAATCTCTCCCTGGAAATTTAAGTTGTTT-3′; reverse, 5′-TCTAGATTACACAGTTAAGTCACACATCTTATTCTC-3′. JAK2: forward, 5′-GGATCCATGGGAATGGCCTGCCTTACAATGACAGAAATG; reverse, 5′-GCGGCCGCTCACGCAGCTATACTGTCCCGGATTTGATC. SH2B1: forward, 5′-AGATCTATGAATGGTGCCCCTTCCCCAGAGGAT; reverse, 5′-CTCGAGTTACACAGTCACTCGCTCCGAAGGGCA. Amplified products were subcloned into the pTAKN-2 vector (BioDynamics). The cDNA of LepRb was subcloned into the expression vector p3xFLAG-CMV (Sigma-Aldrich) using *Hin*dIII and *Xba*I to yield a FLAG-tagged LepRb (FLAG-LepRb). The cDNA of JAK2 was subcloned into the expression vector pcDNA3.1 (Thermo Fisher Scientific) using *Bam*HI and *Not*I. The cDNA of SH2B1 was subcloned into the expression vector pmCherry-C1 (Clontech) using *Bgl*II and *Xho*I to yield mCherry-tagged SH2B1 (mCherry-SH2B1). PTPRJ constructs (pDisplay-PTPRJ and pGEX-PTPRJ) were described previously^23^. In order to construct pAAV-CAGGS-PTPRJ, the DNA fragment encoding the HA-tagged full-length PTPRJ from the pDisplay-PTPRJ plasmid^24^ was subcloned into pAAV-CAGGS-MCS-WPRE using *Eco*RV. The DNA sequences of all constructs were confirmed by DNA sequencing.

### Cell culture and transfection

The immortalized hypothalamic cell line, mHypoA-2/10, was purchased from CEDARLANE Corporation. mHypoA-2/10 and HEK293T cells were grown in Dulbecco’s Modified Eagle’s Medium (DMEM)/F-12 medium (1:1, Nissui) supplemented with 10% fetal bovine serum and antibiotics (100 U/ml penicillin and 0.1 mg/ml streptomycin). Transfection to 293 T cells was performed using LipofectAMINE LTX PLUS (Thermo Fisher Scientific) according to the manufacturer’s protocol. Twenty-four hours after transfection, cells were transferred into serum-free DMEM/F-12 for an additional 24 h. Serum-starved cells were stimulated for 15 min with 50 ng/ml leptin (WAKO), and cell lysates were prepared as previously described^24^.

### Phosphatase assay with phosphopeptides

Phosphopeptides were custom-synthesized by Biologica. Phosphatase assays were performed as previously described^24^. The amount of phosphate released from phosphopeptides was measured as malachite green-ammonium molybdate phosphate complex at absorbance 595 nm.

### Western blotting

Western blotting was performed following a previously described protocol^24^. Briefly, blotted membranes were reacted with specific primary antibodies and peroxidase-linked secondary antibodies, and proteins were visualized by chemiluminiscence using an ECL Reagent (PerkinElmer). The luminoimage analyzer LAS-5000 (Fujifilm) was used for detection.

### Intracerebroventricular (i.c.v.) administration

Mice were anesthetized and placed in a stereotaxic apparatus. The skull was exposed and the i.c.v. cannula was implanted into the left lateral ventricle (0.4 mm posterior to the bregma, 1.0 mm lateral relative to the midline, and 2.3 mm below the surface of the skull). One week after the implantation, the correct placement of the cannula was confirmed by dipsogenic responses after administration of angiotensin II (25 ng, 1 μl). After habituation, mice were treated with leptin (2 μg or 500 ng, 2 μl) or vehicle i.c.v. administration 1 h before the onset of the dark phase. Food intake and body weight were measured 24 h later. In the assessment of STAT3 phosphorylation levels, mice were fasted for 18 h and then injected with leptin (2 μg, 2 μl) or vehicle. After 30 min, MBH was isolated and processed for immunoblot analyses with antibodies for pSTAT3 and STAT3. In order to prepare hypothalamic samples of *ob*/*ob* mice, mice were treated with leptin (2 μg, 2 μl) or vehicle by i.c.v. administration 1 h before the onset of the dark phase for 3 days. MBH was isolated 16 h after the last administration.

### Real-time PCR

Total RNA from tissues was extracted using Trizol reagent (Thermo Fisher Scientific) according to the manufacturer’s instructions. Equal amounts of total RNA were reverse transcribed to cDNA using the PrimeScript RT regent Kit with a DNA eraser (Takara). We confirmed the removal of genomic DNA by PCR without an RT reaction. Primers were designed using the Perfect Real Time support system (Takara) as follows. *Ptprj*: 5′-CACAGCTGAGATAGCCGAGAACA-3′ (forward) and 5′-GTCGAATGGGTCTGGACTGAAAG-3′ (reverse).

*Ptp1b*: 5′-TGGATCTCAGACATTCCACACTCAC-3′ (forward) and 5′-AGCTGCCTTGCTTCCAGTCC-3′ (reverse).

*Gapdh*: 5′-TGTGTCCGTCGTGGATCTGA-3′ (forward) and 5′-TTGCTGTTGAAGTCGCAGGAG-3′ (reverse).

Gene expression was quantified using a StepOnePlus Real Time PCR System (Thermo Fisher Scientific) and SYBR Premix Ex Taq II (Takara). The expression levels of mRNA were normalized to the *Gapdh* mRNA content.

### Virus Injection into ARC

The AAV vectors, AAV-DJ-PTPRJ (titer, 3.5 × 10^12^ genomic copies (GC)/ml) and AAV-DJ-EGFP (titer, 3.1 × 10^12^ GC/ml), were prepared using the AAV Helper Free Packaging System (Cell Biolabs) as described previously^60^. Regarding virus injections, male C57BL6/J mice fed ND at 12–16 weeks of age were deeply anesthetized with pentobarbital-xylazine and mounted onto a stereotaxic apparatus. After exposing the skull via a small incision, a small hole was drilled for injections. Bilateral injections to ARC were targeted 1.5 mm posterior to the bregma, 0.2 mm lateral to the midline, and 5.8 mm deep from the brain surface. A pulled-glass pipette was inserted into the brain, and the virus solution was injected with a microsyringe pump (Ultra Micro Pump III, World Precision Instruments) at a rate of 0.1 μl/min for 5 min. After the glass micropipette was withdrawn, i.c.v. cannulation was performed as described above. Animals were allowed to recover for at least 1 week and then subjected to experiments of leptin administration. All stereotaxic injection sites were verified by immunohistochemistry at the end of each experiment. When virus infection to ARC was unsuccessful, data obtained from these mice were excluded from analyses.

### Statistical analysis

Statistical analyses were performed using the Student’s *t*-test, ANOVA, followed by Scheffe’s post hoc test, and ANCOVA^59^ using SigmaPlot (version 13; Systat Software). Results are presented as the mean ± s.e.m. *P* < 0.05 was considered to be significant.

## Electronic supplementary material

Supplementary Information
