# Supplementary material for: PTPRJ Inhibits Leptin Signaling, and Induction of PTPRJ in the Hypothalamus Is a Cause of the Development of Leptin Resistance
Source: Sci Rep. 2017 Sep 14;7:11627. doi: 10.1038/s41598-017-12070-7 (PMC5599550; doi:10.1038/s41598-017-12070-7)
Supplement: Supplementary file 1 — Supplementary Information [file 41598_2017_12070_MOESM1_ESM.pdf]

Supplementary Information

PTPRJ Inhibits Leptin Signaling, and Induction of PTPRJ in the Hypothalamus Is a Cause of the Development of Leptin Resistance

Takafumi Shintani, Satoru Higashi, Ryoko Suzuki, Yasushi Takeuchi, Reina Ikaga, Tomomi Yamazaki, Kenta Kobayashi and Masaharu Noda

Supplementary Figure S1. Analyses of food intake and energy expenditure by *Ptprj*-KO mice.

Supplementary Figure S2. Female *Ptprj*-KO mice are lean on ND.

Supplementary Figure S3. Protein expression in the MBH of *Ptprj*-KO mice.

Supplementary Figure S4. Suppression of the interaction between SH2B1 and JAK2 by the coexpression of PTPRJ.

Supplementary Figure S5. Expression of NPY and α-MSH in *Ptprj*-KO mice.

Supplementary Figure S6. Comparison of the pSTAT3/STAT3 ratio between ND-fed and HF/HSD-fed groups.

Supplementary Figure S7. Bilateral injection of AAV-PTPRJ vectors into the ventral hypothalamus.

Supplementary Figure S8. Expression of *Ptp1b* in the various tissues.

Supplementary Figure S9. Full-length blots for Figure 3A.

Supplementary Figure S10. Full-length blots for Figure 3C.

Supplementary Figure S11. Full-length western blots for Figure 3D.

Supplementary Figure S12. Full-length blots for Figure 4C.

Supplementary Figure S13. Full-length blots for Figure 5A-C.

Supplementary Figure S14. Full-length blots for Supplementary Figure S3.

Supplementary Figure S15. Full-length blots for Supplementary Figure S4.

Supplementary Figure S16. Full-length blots for Supplementary Figure S6 and S7B.

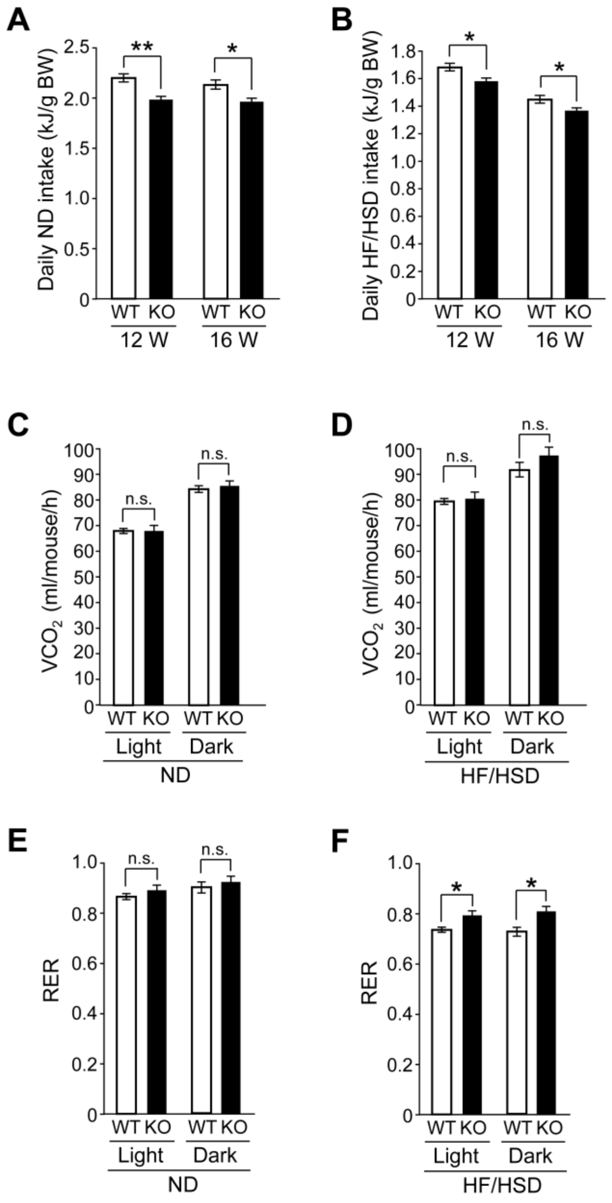

Supplementary Figure S1. Analyses of food intake and energy expenditure by *Ptprj*-KO mice. (A) Daily ND intake by WT and KO mice during 12 and 16 weeks of age (n = 10 each). (B) Daily HF/HSD intake by WT and KO mice during 12 and 16 weeks of age (n = 10 each). (C), (D) Carbon dioxide production (VCO_2), and (E), (F) the respiratory exchange rate (RER) were analyzed during a 48-h period in WT and KO mice fed ND or HF/HSD at 16 weeks of age (n = 8 each). Data were expressed as the mean ± s.e.m. Data were analyzed by the Student's t-test (A,B) or ANCOVA (C-F): *$P < 0.05$; **$P < 0.01$; n.s., not significant.

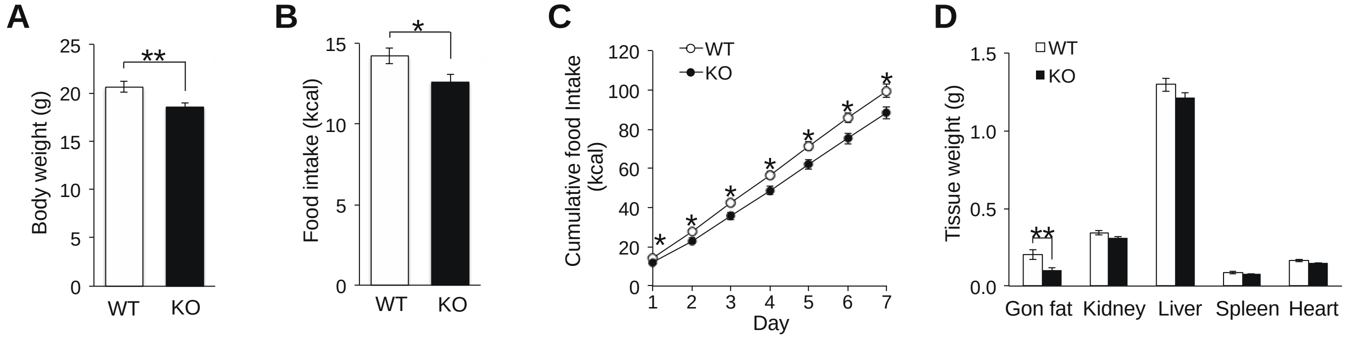

Supplementary Figure S2. Female *Ptprj*-KO mice are lean on ND. (A) Body weight, (B) daily food intake, and (C) cumulative food intake by female WT and *Ptprj*-KO mice (at 10 weeks of age, n = 10 each). (D) Tissue weights of female WT and KO mice on ND (at 12 weeks of age, n = 8 each). Data expressed are as the mean ± s.e.m. Data were analyzed by the Student's *t*-test and ANOVA: *$P < 0.05$, **$P < 0.01$.

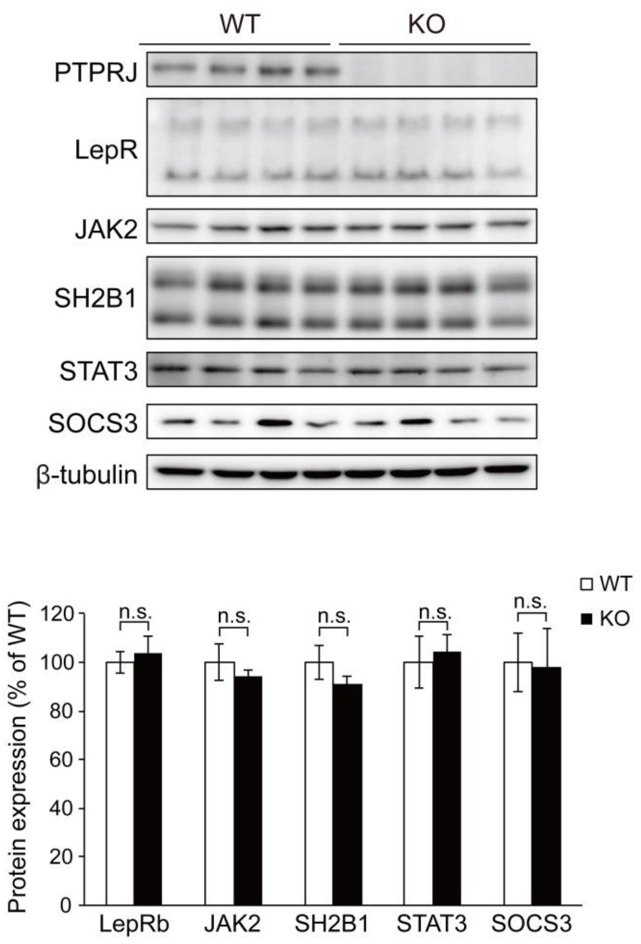

Supplementary Figure S3. Protein expression in the MBH of *Ptprj*-KO mice. Western blots of PTPRJ, LepR, JAK2, SH2B1, STAT3, SOCS3, and β-tubulin in the MBH of WT and *Ptprj*-KO mice (n = 4 each). Each lane indicates the sample from an individual mouse. Western blotting using the LepR antibody exhibits two bands, which may correspond to the long (LepRb) and short (LepRa) isoforms, respectively. The SH2B1 antibody also detects the two isoforms of SH2B1. Full-length blots are presented in Supplementary Fig. S14. The lower graph shows a summary of the expression levels of the proteins (n = 4 each). Data were normalized to the protein levels of WT mice. Data are expressed as the mean ± s.e.m. n.s., not significant.

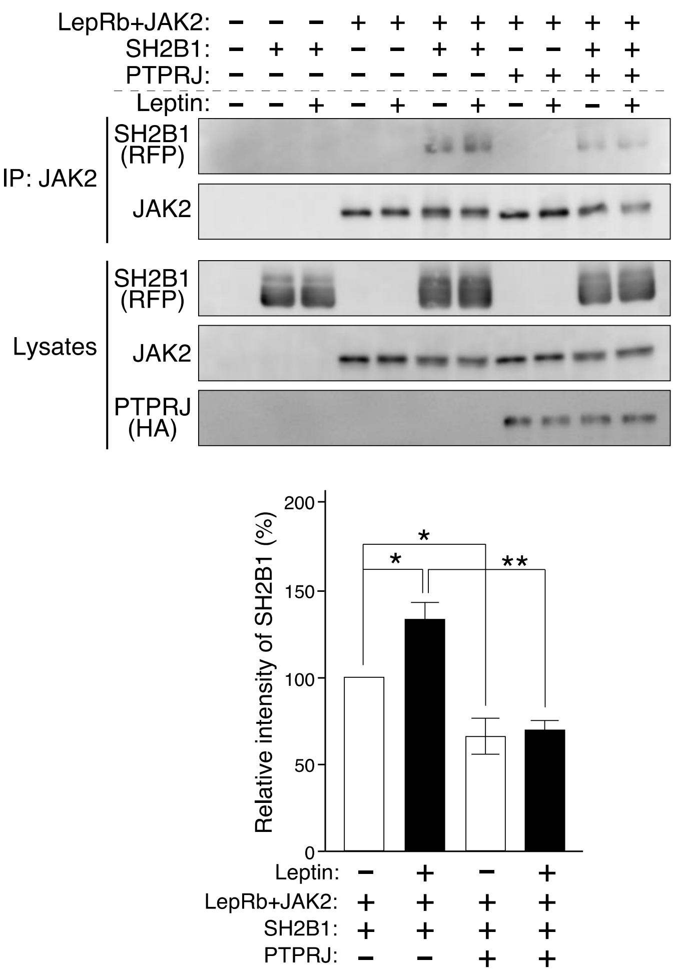

Supplementary Figure S4. Suppression of the interaction between SH2B1 and JAK2 by the co-expression of PTPRJ. Cell lysates were immunoprecipitated with anti-JAK2, and co-immunoprecipitated SH2B1 proteins were detected with the anti-RFP antibody. Full-length blots are presented in Supplementary Fig. S15. The lower graph shows summaries of the amount of co-immunoprecipitated SH2B1 with JAK2 (n = 4). Data were normalized to the protein levels of immunoprecipitated JAK2 and shown as relative values of cells expressing LepRb and JAK2 without the leptin stimulation. Data are expressed as the mean ± s.e.m. $*P < 0.05$ and $**P < 0.01$ indicate a significant difference between the indicated pairs (ANOVA followed by Scheffe's post-hoc test).

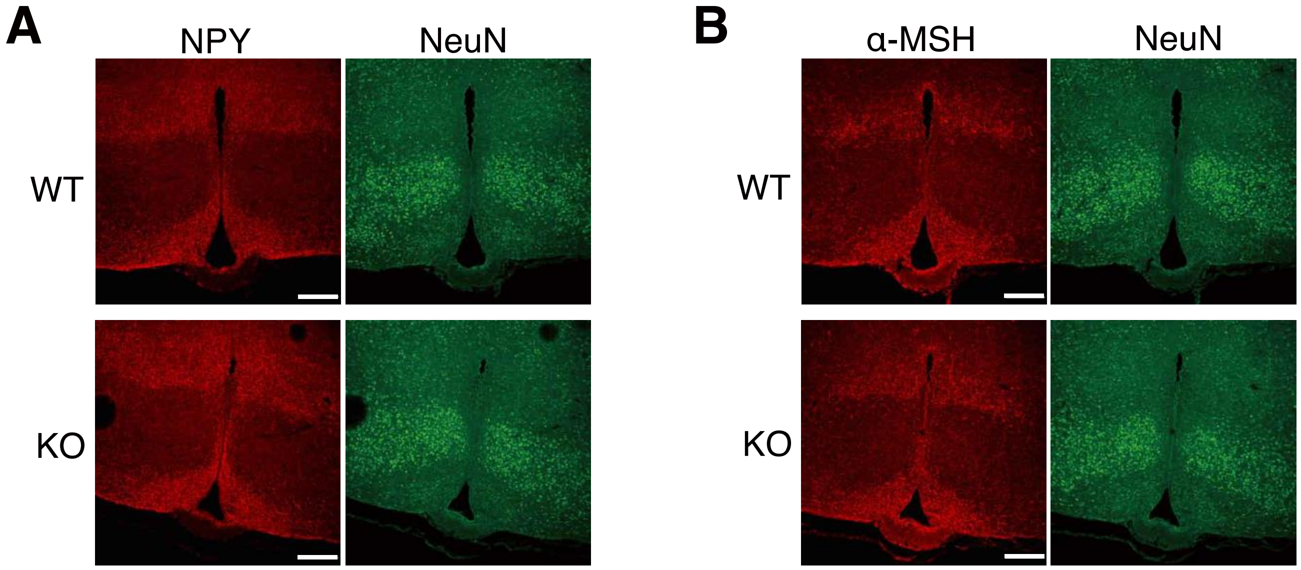

Supplementary Figure S5. Expression of NPY and α-MSH in *Ptprj*-KO mice. (A) Immunohistochemistry of NPY(red) and NeuN (green) in the MBH of WT and *Ptprj*-KO mice. (B) Immunohistochemistry of α-MSH (red) and NeuN (green) in the MBH of WT and *Ptprj*-KO mice. Scale bar: 200 μm.

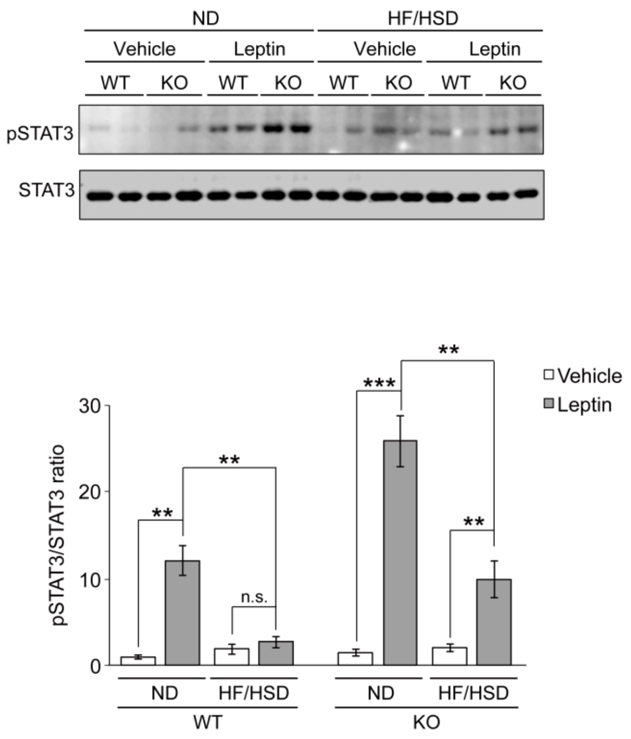

Supplementary Figure S6. Comparison of the pSTAT3/STAT3 ratio between ND-fed and HF/HSD-fed groups. Western blot analyses of pSTAT3 and total STAT3 in the MBH lysate prepared from WT and KO mice fed ND or HF/HSD (n = 7 each). Mice were fasted for 18 h, injected with leptin (2 μg) or vehicle, and killed 30 min later. The upper panel shows a representative image of Western blotting. Data are individually composed of different mouse samples. The lower graph shows the summary of quantitative analyses, which represents the relative ratio of pSTAT3/STAT3. Full-length blots are presented in Supplementary Fig. S16. Data are expressed as the mean ± s.e.m. **P* < 0.05, ***P* < 0.01, and ****P* < 0.001 represent significant differences between the indicated pairs (ANOVA followed by Scheffe’s post hoc test).

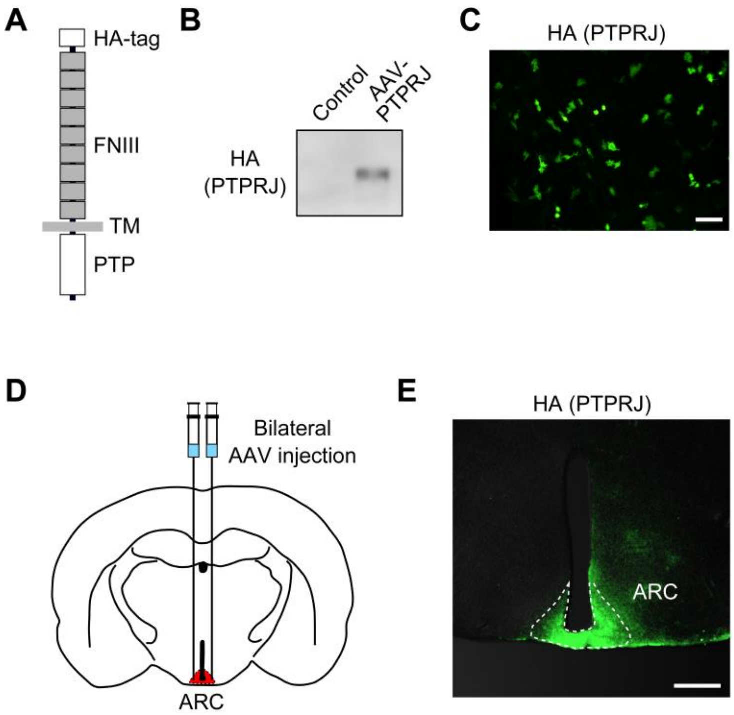

Supplementary Figure S7. Bilateral injection of AAV-PTPRJ vectors into the ventral hypothalamus. (A) Schematic representation of the PTPRJ protein structure expressed by AAV-PTPRJ vectors. HA-tag, hemagglutinin-tag; FNIII, fibronectin type III repeat domains; TM, transmembrane region; PTP, PTP domain. (B) Western blotting of lysates of Neuro2a cells infected with AAV-PTPRJ. Lysates from non-infected (control) and AAV-PTPRJ-infected cells were analyzed with the HA antibody. Full-length blots are presented in Supplementary Fig. S16. (C) Immunostaining of Neuro2a cells infected with AAV-PTPRJ. Cells were immunostained with the HA antibody. (D) Schematic representation of the bilateral injection of AAV-PTPRJ into the ventral hypothalamus. ARC, arcuate nucleus. (E) Representative image of HA-PTPRJ expression in the ventral hypothalamus. A hypothalamic slice infected with AAV-PTPRJ was immunostained with the HA antibody. Scale bar: (**C**) 100 μm and (**E**) 200 μm.

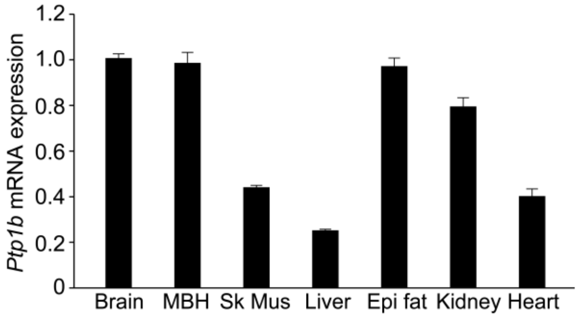

Supplementary Figure S8. Expression of *Ptp1b* in various tissues. Quantitative real-time RT-PCR analyses of *Ptp1b* mRNA levels in various tissues (at 14 weeks of age, n = 4 each). Data are normalized to the expression level of *Gapdh* in each tissue and shown as relative values. Brain, whole brain; MBH, mediobasal hypothalamus; Sk Mus, skeletal muscle; Epi fat, epididymal fat.

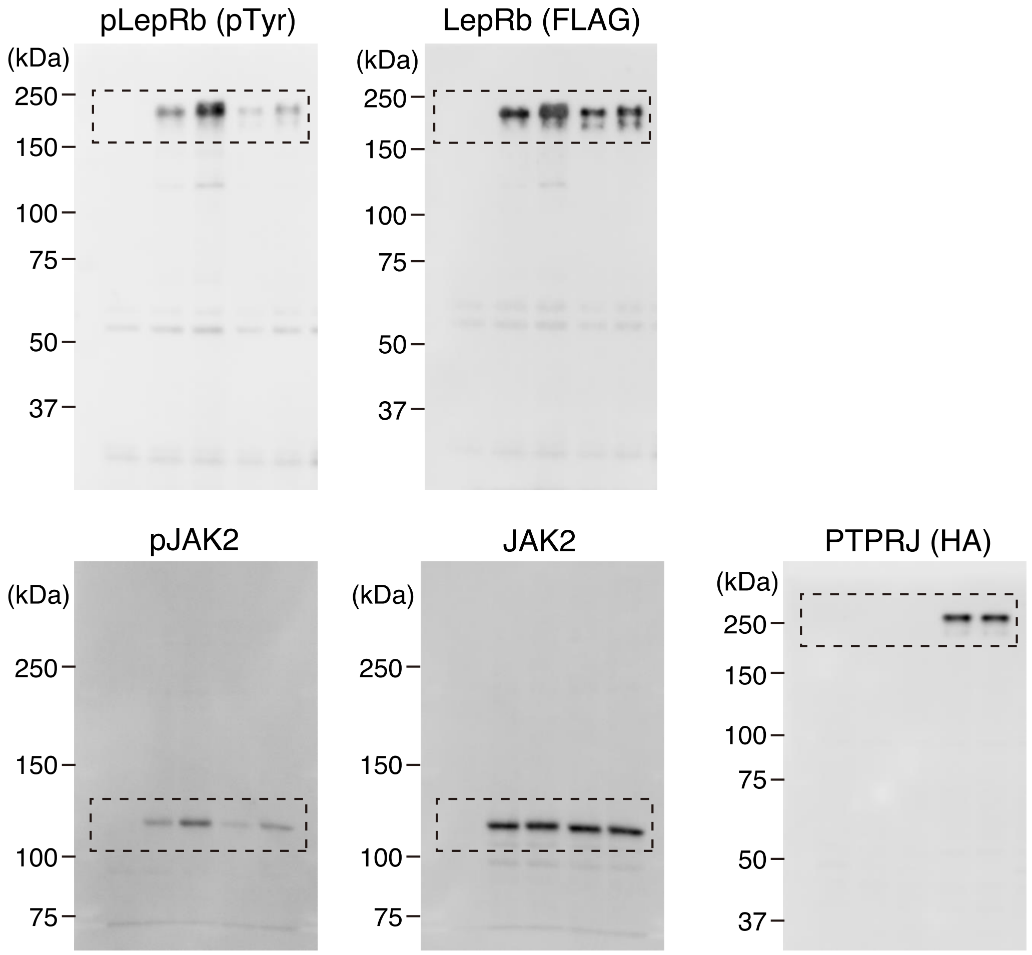

Supplementary Figure S9. Full-length blots for Figure 3A.

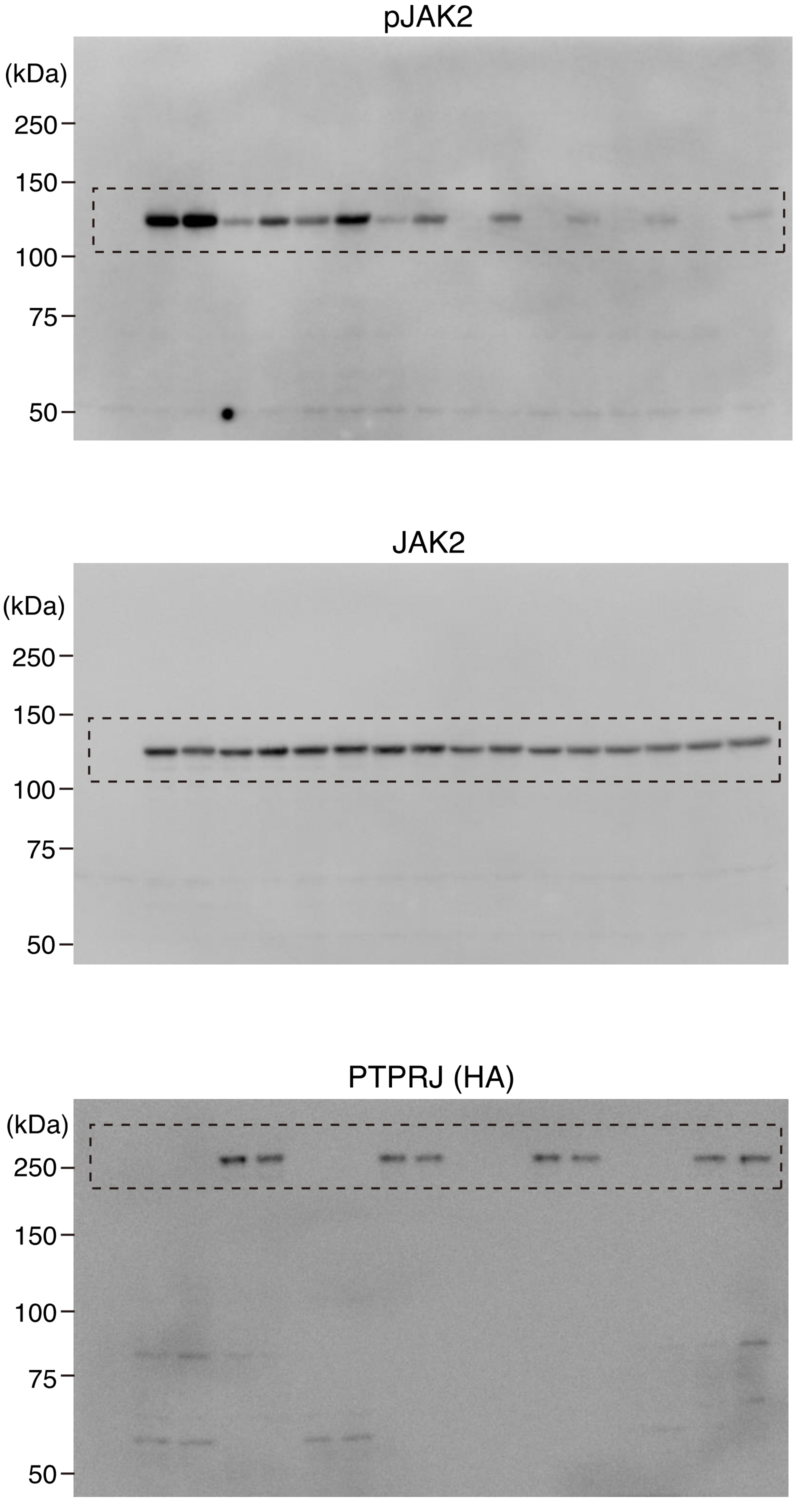

Supplementary Figure S10. Full-length blots for Figure 3C.

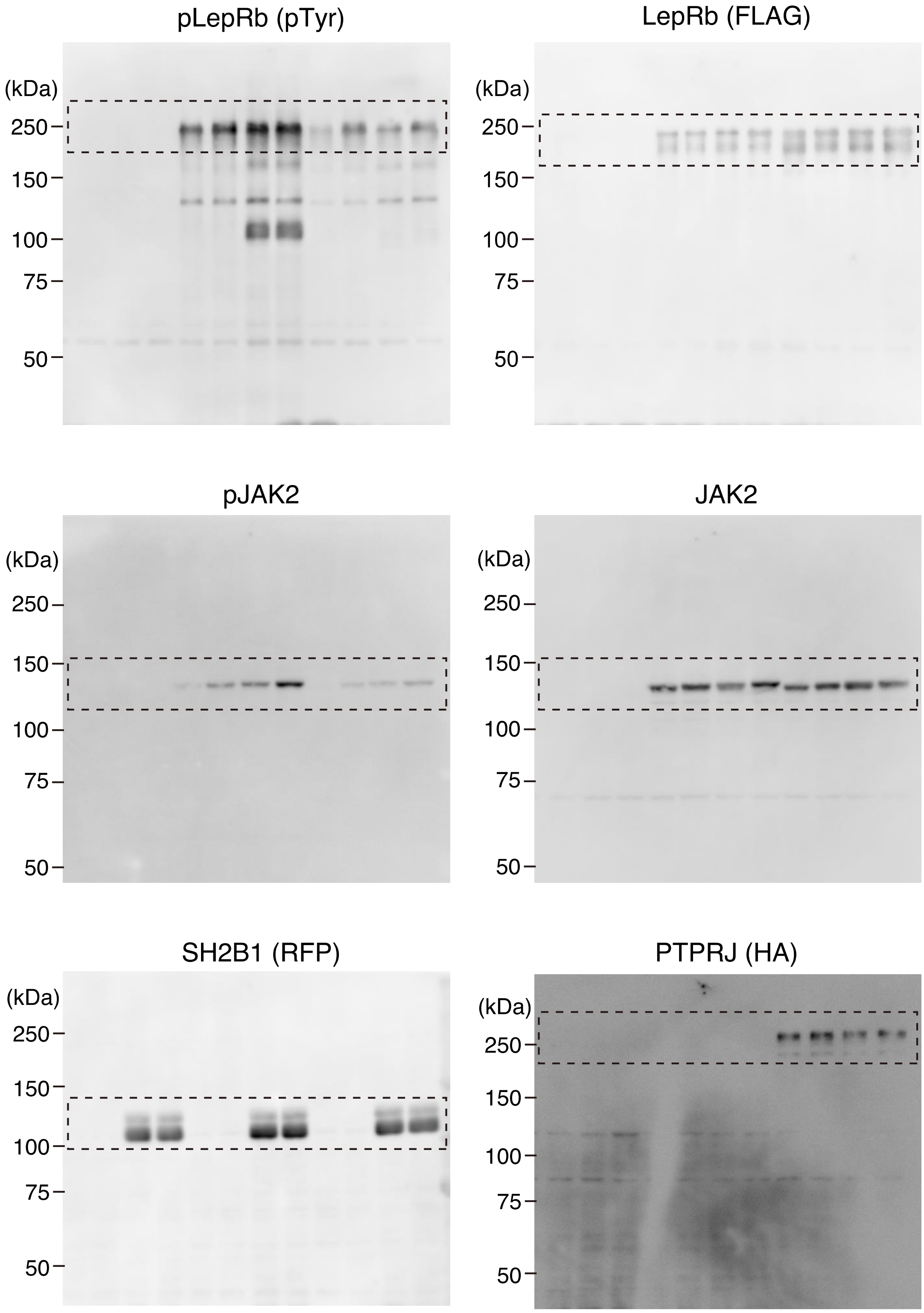

Supplementary Figure S11. Full-length blots for Figure 3D.

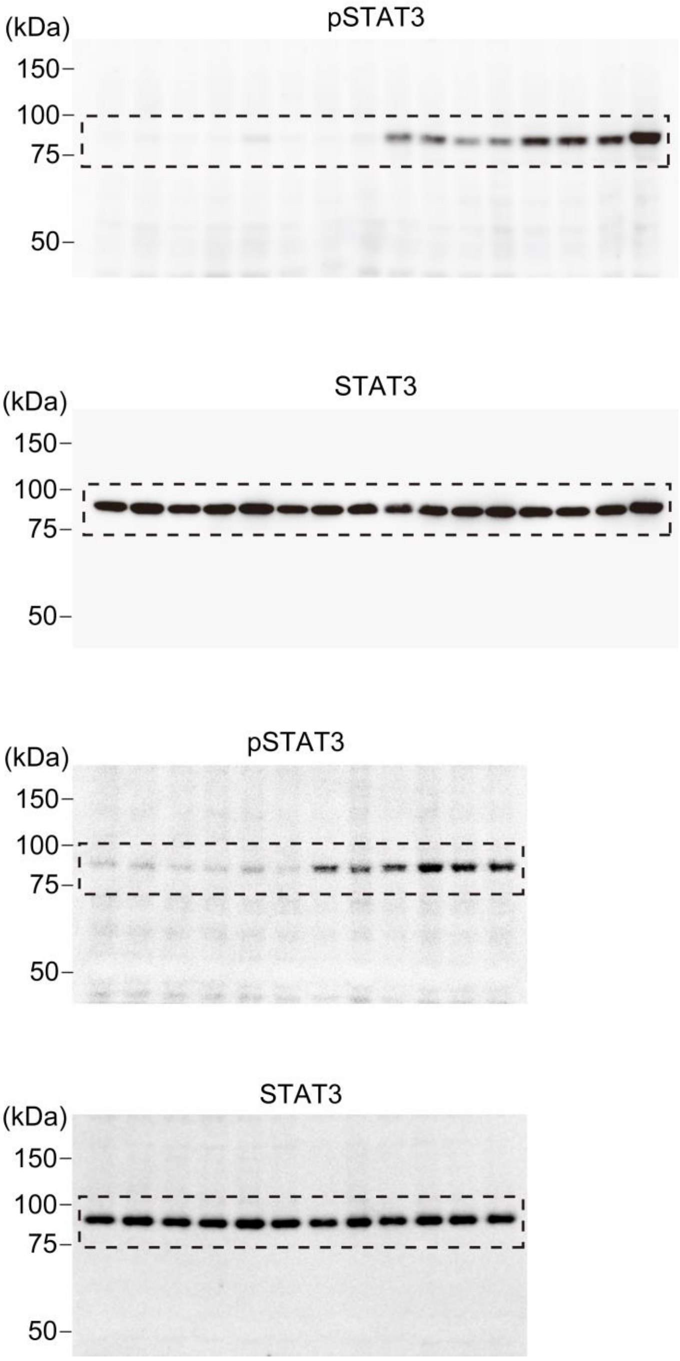

Supplementary Figure S12. Full-length blots for Figure 4C.

Fig. 5A

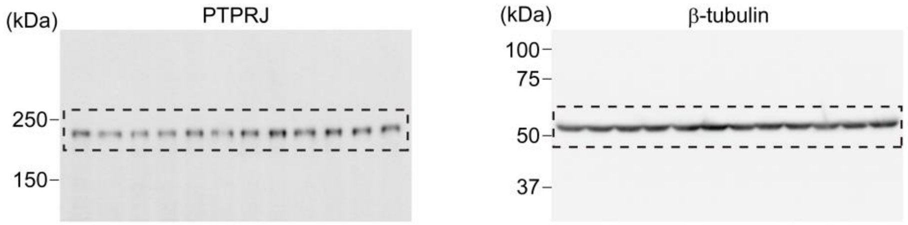

Fig. 5B

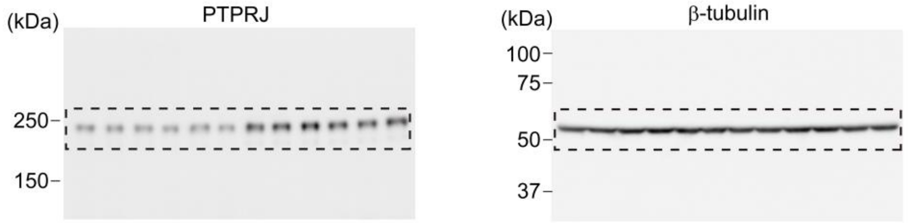

Fig. 5C

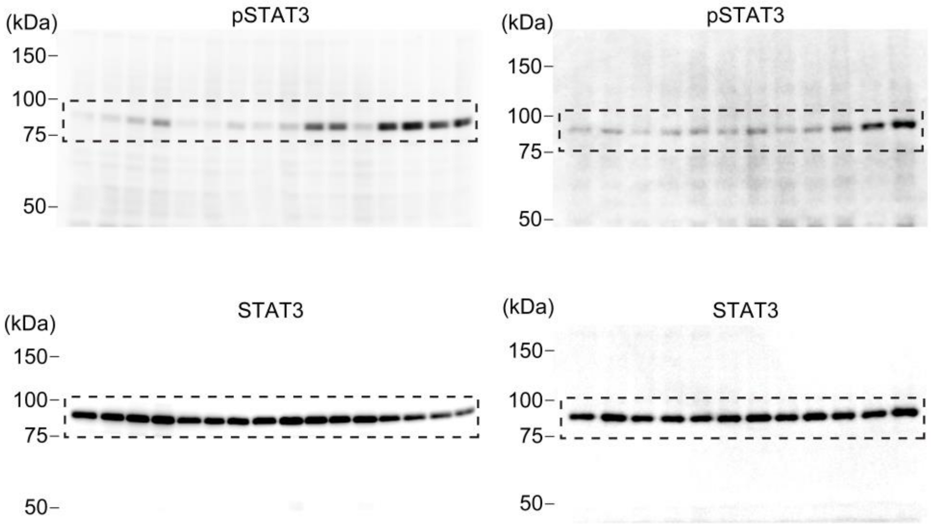

Supplementary Figure S13. Full-length blots for Figure 5A-C.

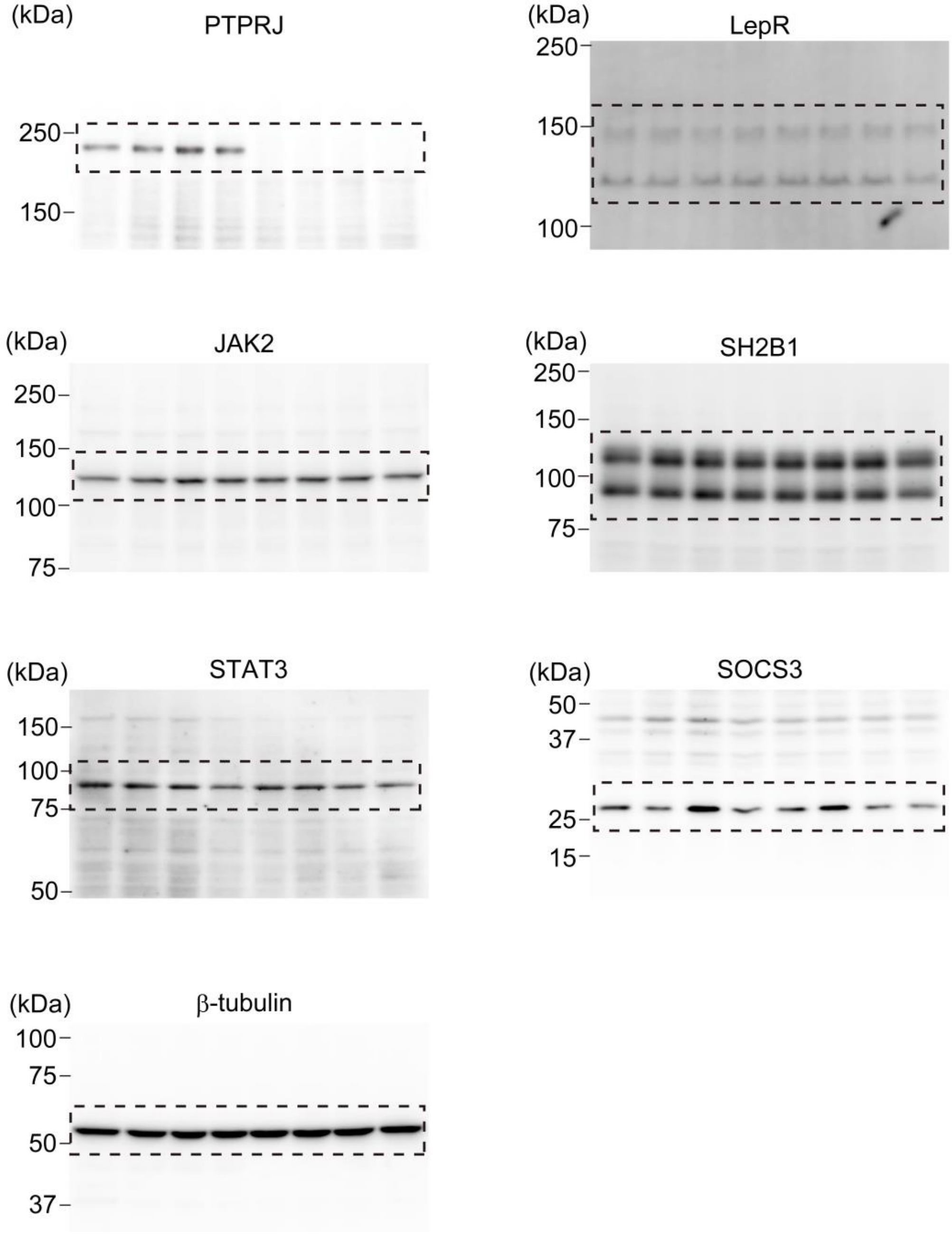

Supplementary Figure S14. Full-length blots for Supplementary Figure S3.

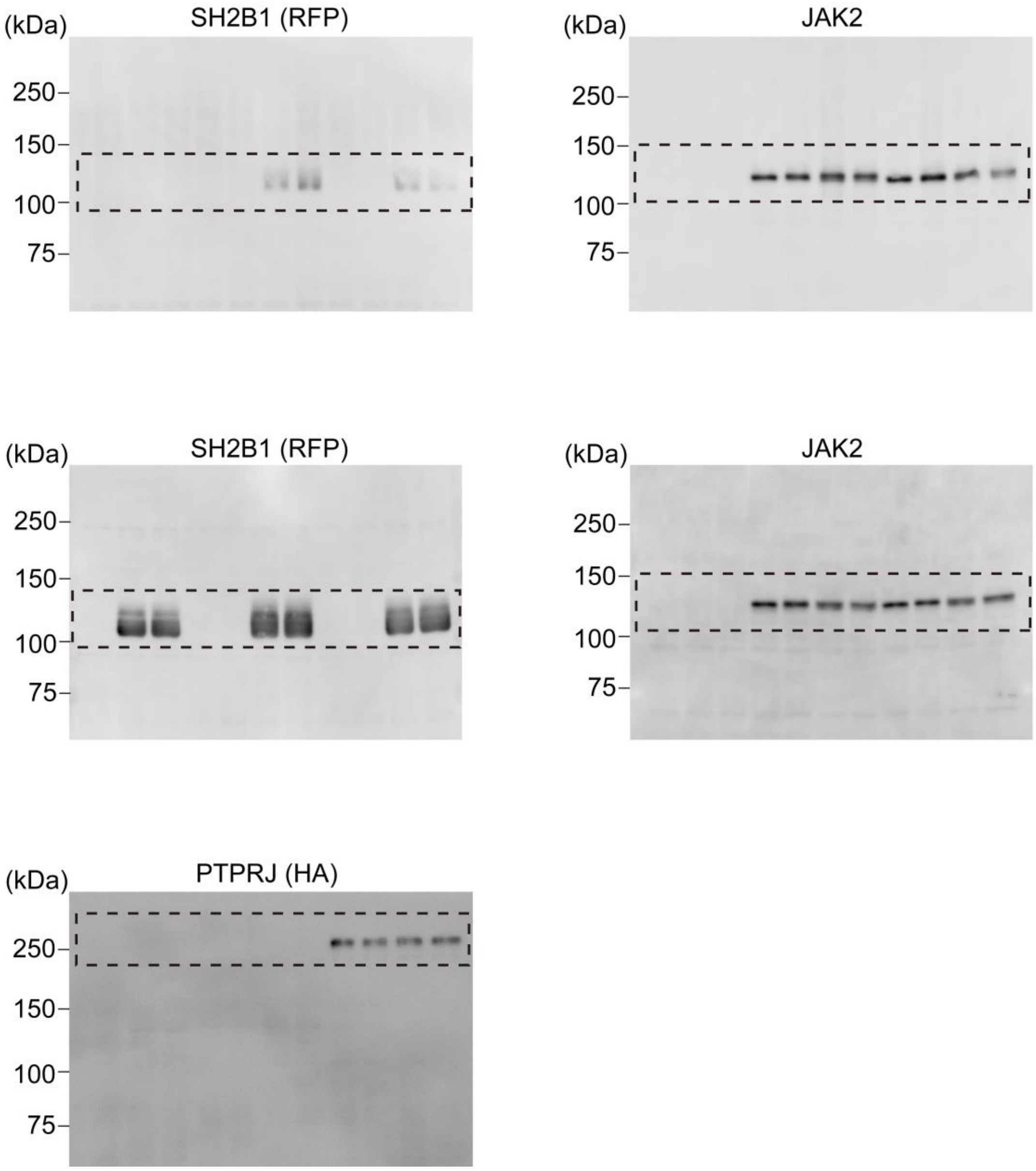

Supplementary Figure S15. Full-length blots for Supplementary Figure S4.

Fig. S6

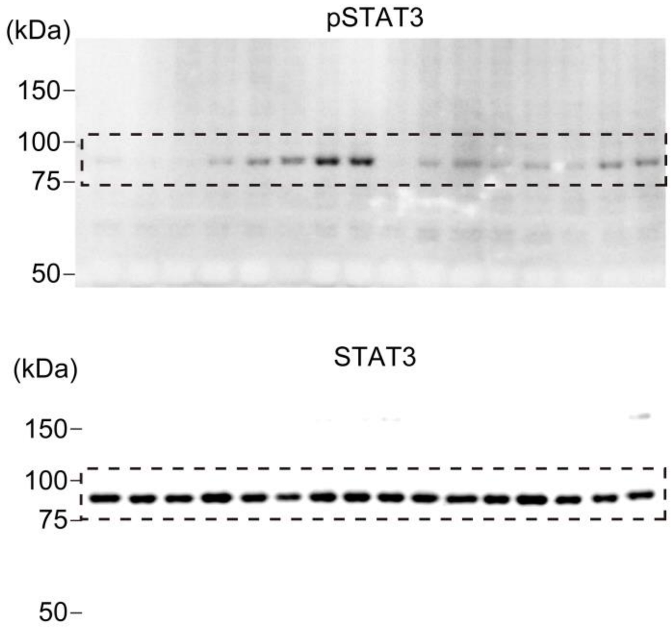

Fig. S7B

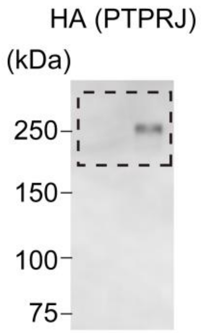

Supplementary Figure S16. Full-length blots for Supplementary Figure S6 and S7B.
